# Almost automorphic and bijective factors of substitution shifts

**DOI:** 10.1007/s00605-024-02053-y

**Published:** 2025-01-16

**Authors:** Alvaro Bustos-Gajardo, Johannes Kellendonk, Reem Yassawi

**Affiliations:** 1 School of Mathematics and Statistics, The Open University, Walton Hall, Kents Hill, Milton Keynes, MK7 6AA UK; 2 Facultad de Matemáticas, Pontificia Universidad Católica de Chile, Marcoleta 367, 8320165 Santiago, Chile; 3 Institut Camille Jordan, Université Lyon-1, 43 Bd du 11 Novembre 1918, 69100 Villeurbanne, France; 4 School of Mathematical Sciences, Queen Mary University of London, Mile End Road, London, E14NS UK

**Keywords:** Topological factors, Almost automorphic, Substitutions, Green’s relations, 37B02, 37B10, 37B52, 20M10, 20M35.

## Abstract

In this article we completely characterise constant length substitution shifts which have a proper almost automorphic factor, or which have a bijective substitution factor such that the factor map is injective on at least one point. Our approach is algebraic: we characterise these dynamical properties in terms of a finite semigroup defined by the substitution. We characterise the existence of almost automorphic factors in terms of Green’s ${\mathcal {R}}$-relation and the existence of bijective factors in terms of Green’s ${{\mathcal {L}}}$-relation. Our results are constructive.

## Introduction

We are interested in the existence of factors with specific properties for shifts defined by substitutions of constant length. Here, by dynamical system (*X*, *T*) we mean a homeomorphism *T* acting on a compact metric space *X*. If (*X*, *T*) and (*Y*, *S*) are two dynamical systems and $F:X\rightarrow Y$ is a continuous surjective map such that $F\circ T = S\circ F$ then (*Y*, *S*) is called a factor of (*X*, *T*), (*X*, *T*) is called an extension of (*Y*, *T*), and *F* is called the factor map. The factor map is *almost one-to-one* if *Y* has a dense orbit of points, each of which have a unique pre-image; if this is the case we say that (*X*, *T*) is an *almost one-to-one extension* of (*Y*, *S*).^1^ (*X*, *T*) is called *almost automorphic* if it is an almost one-to-one extension of its maximal equicontinuous factor. If an almost automorphic system is not equicontinuous we call it properly almost automorphic. We are interested in characterising two different scenarios regarding a shift $(X_\theta ,\sigma )$ of a constant length substitution $\theta$:

1. $(X_\theta ,\sigma )$ has a proper almost automorphic factor, and
2. $(X_\theta ,\sigma )$ is *almost bijective*, by which we mean that it is an almost one-to-one extension of a *bijective* substitution shift.

In the first scenario, looking for almost automorphic factors which are shift factors already solves the problem. This is because the substitution systems we consider have odometers as maximal equicontinuous factors, and a proper almost one-to-one extension of an odometer is necessarily conjugate to a shift [7, Theorem 6.4]. By [16, Theorem 22], these shift factors must be substitution shifts. So the first scenario boils down to characterising aperiodic primitive substitutions of constant length which have a factor given by a primitive aperiodic constant length substitution shift with a coincidence (in the sense of Dekking). We also mention Martin’s theorem [15, Thm.8.11] which implies that, if a dynamical system (*X*, *T*) has a proper almost automorphic factor then it also has a proper almost automorphic factor which is an almost one-to-one extension of the maximal equicontinuous factor of (*X*, *T*).

Before we explain our methods let us indicate why the study of factors of the type above is interesting beyond the pure interest in them. One reason is simply that certain properties of dynamical systems–and here we think in particular of spectral properties–pass over to their extensions. For instance, it is commonly believed that bijective substitution shifts should have a singular component in their maximal spectral type, and this property would then be true also for substitutions which factor onto a bijective substitution. Another reason is Veech’s structure theorem for point distal systems [19], which applies to constant length substitution shifts; see Sect. 3.6 for a statement and definitions. One can ask what their $\mathcal{A}\mathcal{I}$-tower structure looks like; our findings give a partial answer to this question. A third reason to study in particular shift factors associated with bijective substitutions is that the latter constitute one of the few families for which an explicit description of the Ellis semigroup exists [12] and since factor maps induce epimorphisms of Ellis semigroups, we obtain information on the Ellis semigroup of $(X_\theta ,T)$ in this way. This will be presented in future work.

We study both scenarios above using a combination of two sets of tools. The first tool is algebraic, the semigroup $S_\theta$ of a substitution $\theta$; see Definition 4. This semigroup has been extensively used in the case when it is a group $G_\theta$, i.e., when the substitution is bijective. For example, $G_\theta$ is used to characterise the automorphism group [13, 16], and, if it is commutative, then $(X_\theta ,\sigma )$ has a singular component in its maximal spectral type [2, 18]. Also, it is a fundamental building block of the Ellis semigroup of a bijective substitution [12]. It is interesting that we use Green’s ${\mathcal {R}}$ relation to prove Theorem 1, and Green’s ${\mathcal {L}}$ relation to prove Theorems 2 and 3.

The second set of tools is classical and involves building topologically conjugate versions of $(X_\theta , \sigma )$ using *collaring* and *k-shifting* (Definitions 1 and 2). In particular, collaring allows us to control the radius of a putative factor map $F:X_\theta \rightarrow Y$, and *k*-shifting allows us to compose *F* with a “translation". We use both these constructions to limit and manipulate possible factor maps, and this results in theorems whose proofs are constructive.

Let $\theta ^{(-l,r)}$ denote the $(-l,r)$-collaring of $\theta$, which is the model of $\theta$ that we work with if $F:X_\theta \rightarrow Y$ has left and right radius *l* and *r*. To state our first result, we distinguish between different families of factor maps. The simplest factor maps are those induced by *inner encodings*; see Definition 3. The factor map $F:X_\theta \rightarrow X_\eta$ induced by the inner encoding can be characterised by the fact that it has radius zero and sends fixed points of the substitution $\theta$ to fixed points of $\eta$. Inner encodings define partitions of the alphabet ${\mathcal {A}}_\theta$ of the substitution (Lemma 3). Conversely, there is a natural way to define a partition ${\mathcal {P}}_\theta$, which we call *the coincidence partition of* $\theta$ (Definition 5), and which yields an inner encoding of $\theta$, called the *canonical inner encoding of* $\theta$. Its definition involves Green’s ${\mathcal {R}}$-relation on the kernel of $S_\theta$. The shift of this inner encoding is almost automorphic; however one cannot guarantee that it is infinite. Our first result, consisting of Theorem 9 and Corollary 4, is

### Theorem 1

Let $\theta$ be a primitive, constant length, aperiodic substitution, with pure base ${\tilde{\theta }}$. Then $\theta$ has a proper almost automorphic factor if and only if the canonical inner encoding of $\tilde{\theta }^{(-1,1)}$ is aperiodic.

The beauty of this result is that it is quite simple to verify its conditions for a fixed substitution. Note that as a corollary, we can show that there exist substitution shifts for which the maximal *tame* factor, [8], equals the maximal equicontinuous factor. For, a tame factor must be almost automorphic [11], and with Theorem 1, we can give many examples of substitution shifts with no proper almost automorphic factor.

We discuss prior work concerning Theorem 1. In [15], Martin gives a necessary and sufficient condition for a bijective substitution shift to be an extension of a proper almost automorphic factor in [15, Theorem 8.08], and his condition translates to our condition that the minimal sets for the collaring $\theta ^{(0,1)}$ of the substitution form a partition; see Theorem 11. Later, in his thesis, Herning [10] re-approaches the question of existence of a proper almost automorphic factor; it seems he was unaware of Martin’s work. He answers this question in the negative by finding bijective substitutions that do not have a proper almost automorphic factor. In [10, Theorem 4.24], Herning characterises bijective length-*p* substitutions of prime length that have a proper almost automorphic factor. Again, once translated, his characterisation is very similar to ours and Martin’s. Our Theorem 1 extends these results to characterise when any primitive constant length substitution, not just bijective, has a proper almost automorphic factor.

Our second result characterises, in terms of the semigroup $S_\theta$, when the substitution shift $(X_\theta ,\sigma )$ is an almost one-to-one extension of a bijective substitution shift. We make use of the fact that $S_\theta$ admits a kernel, i.e., a minimal bilateral ideal, which is a union of minimal left ideals. The *column rank* of $\theta$ is the rank of any element in the kernel of $S_\theta$. We first show the following, which is Theorems 13 and 14 of this article.

### Theorem 2

Let $\theta$ be a constant length substitution with column rank *c* and height *h*. Suppose that $c>h$. The following are equivalent.

1. $(X_\theta , \sigma )$ is an almost one-to-one extension of a bijective substitution shift $(X_\eta , \sigma )$ where $\eta$ is an inner encoding of $\theta$ with an alphabet of *c* letters.
2. The semigroup $S_\theta$ of $\theta$ has a unique minimal left ideal.

Note that if $(X_\theta , \sigma )$ is an almost one-to-one extension of any constant length substitution shift $(X_\eta , \sigma )$ then $\eta$ has to have the same column rank. So the requirement that the alphabet of $\eta$ has *c* letters in (1) comes for free. Therefore, if $\theta$ is aperiodic and $c=1$, there is no such factor as $\eta$ must be aperiodic for the factor map to be almost one-to-one. We also show that in the case that $c=h$, $\eta$ is periodic.

To drop the condition that the bijective factor comes from an inner encoding, let $\theta ^{(+k)}$ be the *k*-shifted extension of $\theta$. This version of $\theta$ is especially useful when one considers factor maps *F* which do not map fixed points of $\theta$ to fixed points. We show

### Theorem 3

Let $\theta$ be an aperiodic primitive constant length-$\ell$ substitution with column rank *c* and height *h*. Suppose that $c>h$. The following are equivalent:

1. $(X_\theta ,\sigma )$ is almost bijective.
2. There exist $0\le n,k\le C$ such that the semigroup $S_{(\theta ^n)^{(+k)}}$ contains a unique minimal left ideal.

Moreover, *C* can be explicitly obtained. However in general it is doubly exponential in $\ell$; see the statement of Theorem 17.

We summarise the contents of this paper. In Sect. 2, we set the background and fix notation. In Sect. 3, we define and study *outer encodings* of a substitution; we use these to prove Theorem 1. In Sect. 4, we prove Theorem 3 in three successive steps. We strive to isolate the requirements on the substitution, noting that generally, purely algebraic results do not need the restriction to primitive aperiodic substitutions. Throughout, we illustrate with examples that demonstrate the subtleties that can occur.

## Preliminaries

### Constant length substitutions

A *length*-$\ell$ *substitution* $\theta$ is an ordered collection of $\ell$ maps, the so-called *column maps* $\theta _m:{{\mathcal {A}}}\rightarrow {{\mathcal {A}}}$, $m=0,\cdots ,\ell -1$, on a finite set ${\mathcal {A}}$ (or ${{\mathcal {A}}}_\theta$ if more precision is needed), its alphabet. The substitution $\theta$ can be understood as a map which associates to a letter $a\in {{\mathcal {A}}}$ the word $\theta (a):=\theta _0(a)\cdots \theta _{\ell -1}(a)$ and to a word $a_1\cdots a_k$ the word

1 $$\begin{aligned} \theta (a_1\cdots a_k) = \theta (a_1)\cdots \theta (a_k), \end{aligned}$$

of length $k\ell$, and to the bi-infinite sequences $\cdots u_{-2} u_{-1} u_0 u_1 \cdots$ the bi-infinite sequence

$$\begin{aligned}\theta (\cdots u_{-2} u_{-1} \cdot u_0 u_1 \cdots ):= \cdots \theta (u_{-2}) \theta ( u_{-1} ) \cdot \theta ( u_{0}) \theta ( u_{1}) \cdots \,.\end{aligned}$$

Here the $\cdot$ indicates the position between the negative indices and the nonnegative indices.

A bi-infinite sequence *u* is $\theta$-*periodic* if $\theta ^k(u)=u$ for some $k\ge 1$. If $k=1$ then we say that *u* is a *fixed point*. By taking a power of $\theta$ if necessary, we will assume that each $\theta$-periodic point is $\theta$-fixed. We say that a finite word is *allowed* for $\theta$ if it appears somewhere in $\theta ^k(a)$ for some $a\in {{\mathcal {A}}}$ and some $k\in {\mathbb {N}}$. The *substitution shift* $( X_\theta , \sigma )$ is the dynamical system where the space $X_\theta$ consists of all bi-infinite sequences all of whose subwords are allowed for $\theta$. We equip $X_\theta$ with the subspace topology of the product topology on ${{\mathcal {A}}}^{\mathbb {Z}}$, making the left shift map $\sigma$ a continuous ${\mathbb {Z}}$-action.

In this article our techniques are a combination of algebraic arguments involving finite semigroups, and dynamical techniques applied to the dynamical system $(X_\theta , \sigma )$ generated by $\theta$. For the algebraic arguments, very few constraints are imposed on $\theta$. For the dynamical arguments, we collect the various properties of substitutions which will play a role. We say that $\theta$ is *primitive* if there is some $k\in {\mathbb {N}}$ such that for any $a,a'\in {\mathcal {A}}$, the word $\theta ^k(a)$ contains at least one occurrence of $a'$. For dynamical arguments, substitutions will mostly be assumed primitive. Primitivity of $\theta$ implies that $X_\theta$ is the shift-orbit closure of any $\theta$-periodic point, and $(X_\theta ,\sigma )$ is minimal. If $\theta$ is primitive, then $X_\theta =X_{\theta ^n}$ for each $n\in {\mathbb {N}}$. Thus, by considering a power of $\theta$ if necessary, we will assume that all $\theta$-periodic points are $\theta$-fixed.

We say that $\theta$ is *aperiodic* if $X_\theta$ does not contain any $\sigma$-periodic sequences. When $\theta$ is primitive, this is the case if and only if $X_\theta$ is an infinite space. We say that $\theta$ is *bijective* if all column maps $\theta _m:{{\mathcal {A}}}\rightarrow {{\mathcal {A}}}$ are bijective.

### The maximal equicontinuous factor of a length-$\ell$ substitution

Let ${\mathbb {Z}}_\ell$ denote the $\ell$-adic integers, i.e., the inverse limit of cyclic groups $\varprojlim {\mathbb {Z}}/\ell ^n{\mathbb {Z}}$. Let ${\mathbb {Z}}_{\bar{\ell },h}:= \varprojlim {\mathbb {Z}}/\ell ^nh{\mathbb {Z}}$ and let $1:= (\cdots , 0,0,1)$; addition in ${\mathbb {Z}}_{\bar{\ell },h}$ is performed with carry. If $\theta$ is primitive and aperiodic, then Dekking’s theorem [5] tells us that $({\mathbb {Z}}_\ell , +1)$ is an equicontinuous factor of $(X_\theta , \sigma )$. Furthermore, there is an *h*, with $0<h<\ell$ and with *h* coprime to $\ell$, such that $({\mathbb {Z}}_{\bar{\ell },h}, +1)$, is the maximal equicontinuous factor of $(X_\theta , \sigma )$. The integer *h* is called the *height* of $\theta$, and we say that $\theta$ has *trivial height* if $h=1$.

We fix the factor map $\pi :X_\theta \rightarrow {\mathbb {Z}}_\ell$ from a primitive aperiodic length-$\ell$ substitution shift $(X_\theta ,\sigma )$ to $({\mathbb {Z}}_\ell ,+1)$ with which we work in this article. We will specify it by requiring $\pi (u)=0$ if and only if *u* is a $\theta$-fixed point. We refer the reader to [5] for details.

Given the substitution $\theta$, the substitution $\theta ^n$ is a length-$\ell ^n$ substitution. If $0\le j \le \ell ^n -1$, we use ${\theta ^n}_j$ to denote its *j*-th column map. The *column rank* of a substitution $\theta$ is defined as the minimal number of distinct letters in the image of a column map of $\theta ^{n}$, for some *n*. In other words,

2 $$\begin{aligned} c=c (\theta ):= \inf _{j,n} \left\{ |{\theta ^n}_j({\mathcal {A}})|: 0\le j < \ell ^n\right\} .\end{aligned}$$

We say that $\theta$ has a *coincidence* if $c=h$. In this case, $(X_\theta ,\sigma )$ is *almost automorphic*, i.e., an almost one-to-one extension of its maximal equicontinuous factor. For details, see [5]. We remark that our notion of column rank agrees with Dekking’s original definition of column number when $h=1$; however it is in this paper more useful to use the present definition, as it is also in [14], where Lemanczyk and Müllner show that *h* divides *c*, and that $(X_\theta , \sigma )$ is a somewhere *c*-to-one extension of $({\mathbb {Z}}_\ell , +1)$.

### Collared and shifted substitutions

It will be necessary to consider collared substitutions and *k*-shifted substitutions of the substitution $\theta$, which yield shifts that are topologically conjugate to $(X_\theta , \sigma )$. The notation we use in the following definition will be useful when we consider recasting factor maps of left radius $l\ge 0$ and right radius $r\ge 0$ as codings.

#### Definition 1

Let $n=(-l,r)$ where $l,r \ge 0$. The *n*-*collared extension of* $\theta$ is the substitution $\theta ^{(n)}$ of the same length whose alphabet consists of the allowed ($r+1+l$)-letter words of $\theta$ and which is given as follows. Given an allowed word $a_{-l}\ldots a_{r}$ compute $a'_{-\ell l} \ldots a'_{\ell (r+1)-1 }:=\theta (a_{-l}\ldots a_{r})$ and set

$$\begin{aligned} {\theta ^{(n)}}_m (a_{-l}\ldots a_{r}):= a'_{m-l}\ldots a'_{m+r}.\end{aligned}$$

If we take $l=r=0$ then we obtain $\theta ^{(n)}=\theta$. If $l=0$ then $\theta ^{(n)}$ is the so-called *r*-sliding block representation of $\theta$; see [18, Section 5.4]. We denote by ${{\mathcal {A}}}^{(2)}\subset {{\mathcal {A}}}^2$ the set of allowed 2-letter words.

#### Definition 2

Let $0\le k\le \ell -1$. The *k*-*shifted extension of* $\theta$ is the substitution $\theta ^{(+k)}$ of the same length whose alphabet is ${{\mathcal {A}}}^{(2)}$ and which is given as follows. Given an allowed word $a_0 a_{1}$, write $\theta (a_0a_1)=a'_0 \ldots a'_{2\ell -1 }$ and set

$$\begin{aligned} {\theta ^{(+k)}}_m (a_0 a_{1} ):= a'_{m+k} a'_{m+k+1}.\end{aligned}$$

Note that $$\theta ^{(+0)}=\theta ^{(0,1)}$$.

### Factors, codes and encoded substitutions

Recall that a factor map between two shifts, $X\subset {\mathcal {A}}^{\mathbb {Z}}$, $Y\subset {\mathcal {B}}^{\mathbb {Z}}$ is a continuous surjective map $F: X\rightarrow Y$ which intertwines the shifts, $F\circ \sigma = \sigma \circ F$. The Curtis-Hedlund-Lyndon theorem states that such a factor map is defined by a local rule, that is, there are integers $l\ge 0$ and $r\ge 0$ and a surjective map $\varphi :{\mathcal {A}}^{r+1+l}\rightarrow {\mathcal {B}}$ so that

$$\begin{aligned} (F(x))_{n}= \varphi (x_{n-l}, \dots , x_{n+r}) \end{aligned}$$

for each $n\in {\mathbb {Z}}$. The quantities *l*, *r* are called the left and right radius of *F* respectively. If *F* is radius zero, i.e., $l=r=0$, then the local rule $\varphi :{\mathcal {A}}\rightarrow {\mathcal {B}}$ of *F* is called a *code*. We use uppercase letters to denote factor maps, and lowercase Greek letters to denote local rules. If we are given a local rule $\varphi$ which defines a factor map, we will denote the factor map by $F_\varphi$.

#### Definition 3

Let $\eta$ and $\theta$ be length-$\ell$ substitutions. We say that $\eta$ is an *inner encoding* of $\theta$ if there exists a surjective map $\beta :{\mathcal {A}}_\theta \rightarrow {\mathcal {A}}_\eta$ which intertwines the column maps of the substitutions, i.e., 
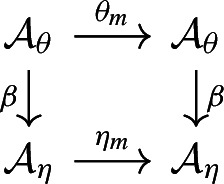


commutes.

If we need more precision then we denote the inner encoding also by the pair $(\eta ,\beta )$. A diagram chase shows that if $\theta$ is primitive then also $\eta$ is primitive. If $(\eta ,\beta )$ is an inner encoding of $\theta$, then $\beta$ is the code of a factor map $F_\beta :X_\theta \rightarrow X_\eta$. However, given an arbitrary code $\beta :{\mathcal {A}}_\theta \rightarrow {\mathcal {A}}_\eta$ for a factor map $F_\beta :X_\theta \rightarrow X_\eta$, it will in general not intertwine the substitutions as above. We will see an example of this below.

#### Lemma 1

Consider a substitution $\theta$ with its $n= (-l,r)$-collaring $\theta ^{(n)}$. Let $\imath :{{\mathcal {A}}}_{\theta ^{(n)}}\rightarrow {{\mathcal {A}}}_\theta$ be given by

3 $$\begin{aligned} \imath (a_{-l}\ldots a_{r}) := a_0 .\end{aligned}$$

Then $(\theta ,\imath )$ is an inner encoding of $\theta ^{(n)}$, and the factor map $F_\imath$ is injective.

#### Proof

Clearly $\imath \circ \theta ^{(n)} = \theta \circ \imath$. To proceed we consider $n=(0,1)$ to not overburden the notation. By definition, every 2-letter word of a sequence $x\in X_{\theta ^{(0,1)}}$ is allowed for $\theta ^{(0,1)}$ and hence of the form $\cdots (a_i a_{i+1}) (a_{i+1} a_{i+2}) \cdots$. Hence

$$\begin{aligned} \cdots (a_i a_{i+1}) (a_{i+1} a_{i+2}) \cdots {\mathop {\mapsto }\limits ^{F_\imath }}\cdots a_i a_{i+1} \cdots \end{aligned}$$

is injective. The argument for different *n* is similar. $\square$

#### Corollary 1

The code ${{\mathcal {A}}}_{\theta ^{(+k)}}\ni a_0 a_{1}{\mathop {\mapsto }\limits ^{\imath }}a_{0}\in {{\mathcal {A}}}_\theta$ gives rise to an injective factor map $F_\imath :X_{\theta ^{(+k)}}\rightarrow X_\theta$ (a conjugacy) which satisfies

$$\begin{aligned} \sigma ^k\circ \theta \circ F_{\imath } = F_{\imath } \circ \theta ^{(+k)}. \end{aligned}$$

#### Proof

All $\theta ^{(+k)}$ have the same alphabet, namely ${{\mathcal {A}}}^{(2)}$, and the same shift space as $\theta ^{(0,1)}$. Indeed, with the notation of Definition 2,

$$\begin{aligned} a_0 a_1 {\mathop {\mapsto }\limits ^{\theta ^{(+k)}}}(a_k'a_{k+1}') ( a_{k+1}' a_{k+2}') \cdots (a_{\ell +k-1}'a_{\ell +k}') \end{aligned}$$

and

$$\begin{aligned} a_0 a_1 {\mathop {\mapsto }\limits ^{\theta ^{(0,1)}}}(a_0'a_1')(a_1' a_2')\cdots (a_{\ell -1}'a_{\ell }) \end{aligned}$$

so that as maps on that shift space we have

$$\begin{aligned} \theta ^{(+k)} = \sigma ^k\circ \theta ^{(0,1)}. \end{aligned}$$

Now the first statement is Lemma 1 for $l=0$ and $r=1$ and the equation follows from $F_\imath \circ \theta ^{(0,1)} = \theta \circ F_\imath$. $\square$

We say that a factor map $F:X_\theta \rightarrow X_\eta$ between two substitution shifts of equal length *preserves the fixed point fibre* if it maps the fixed points of any power of $\theta$ to fixed points of any power of $\eta$.

#### Lemma 2

Let $\eta$ and $\theta$ be length-$\ell$ substitutions and let $\beta :{\mathcal {A}}_\theta \rightarrow {\mathcal {A}}_\eta$ be a code. If $(\eta ,\beta )$ is an inner encoding of $\theta$ then the factor map $F_\beta$ preserves the fixed point fibre. Hence if $(\eta ,\beta )$ is an aperiodic inner encoding of $\theta$, then the equicontinuous factor map $\pi : (X_\theta ,\sigma )\rightarrow {\mathbb {Z}}_\ell$ factors through $F_\beta$. Conversely, if $\theta$ is primitive and $F_\beta : X_\theta \rightarrow X_\eta$ preserves the fixed point fibre, then $(\eta ,\beta )$ is an inner encoding.

#### Proof

Recall that we can assume, by going over to a power of the substitution if needed, that all $\theta$-periodic points of $\theta$ are fixed. Let *v*.*u* be a fixed point of $\theta$ and hence also of $\theta ^m$ for any $m\ge 1$. We denote by $u=u_0 \ldots$ and $\ldots v_{-1} = v$ the right and left infinite parts.

Suppose that $\eta$ is inner encoded by $\beta$, that is, for each *n* and *a*, $\beta (\theta ^n(a))= \eta ^n(\beta (a))$. Then

$$\begin{aligned}F_\beta (v.u) = \lim _n \beta \theta ^n(v_{-1}.u_0)&= \lim _n \eta ^n(\beta (v_{-1}).\beta (u_0)) \\  &= \eta ( \lim _n \eta ^n(\beta (v_{-1}).\beta (u_0))) = \eta (F_\beta (v.u)).\end{aligned}$$

The assumption that $\eta$ is aperiodic implies by Dekking’s theorem that both substitution shifts have $({\mathbb {Z}}_\ell , +1)$ as an equicontinuous factor. Recall that we have fixed these equicontinuous factor maps by requiring that they map the fixed point fibre to $0\in {\mathbb {Z}}_\ell$. As *F* maps the fixed point fibre of $\theta$ to that of $\eta$ we see that the equicontinuous factor map $\pi :X_\theta \rightarrow {\mathbb {Z}}_\ell$ factors through *F* at least for all points in the orbit of a fixed point of $\theta$, and then by minimality for all points of $X_\theta$.

Finally we prove the converse. Suppose that $F_\beta$ sends fixed points to fixed points, so that $F_\beta (v.u)$ is a fixed point of $\eta ^n$ for any $n\ge 1$. If $m < \ell ^n$, then $\beta$ maps the *m*-th letter of *u*, which is ${\theta ^n}_m(u_0)$, to the *m*-th letter of $\beta (u)$, which is ${\eta ^n}_m(\beta (u_0))$. In particular, $\beta$ maps ${\theta ^n}_{m}(\theta _k(u_0))$ to ${\eta ^n}_{m}(\eta _k(\beta (u_0))) = {\eta ^n}_{m}(\beta (\theta _k(u_0)))$. If $\theta$ is primitive all letters arise as ${\theta ^N}_k(u_0)$ for some *N* and *k* showing that $\eta _n(\beta (a)) = \beta (\theta _n(a))$ for all $a\in {{\mathcal {A}}}_\theta$. $\square$

Note that, if $\theta$ is aperiodic, then, for $k>0$, $\theta ^{(+k)}$ cannot be an inner encoding. Indeed, let *u* be a fixed point of $\theta ^{(+k)}$ and suppose that $F_{\imath }$ maps *u* to a fixed point *v* of $\theta$. Applying the equation of Corollary 1 we get

$$\begin{aligned} v = F_{\imath } \circ \theta ^{(+k)}(u) = \sigma ^k\circ \theta \circ F_{\imath } (u) = \sigma ^k(v), \end{aligned}$$

a contradiction.

Any map $\beta :{\mathcal {A}}\rightarrow {\mathcal {B}}$ between sets defines a partition ${{\mathcal {P}}}_\beta = \{ \beta ^{-1}(b): b\in {\mathcal {B}}\}$. We call ${{\mathcal {P}}}_\beta$ the *partition associated to* $\beta$.

#### Lemma 3

Let $\theta$ be a length-$\ell$ substitution on ${\mathcal {A}}$.

1. If $(\eta ,\beta )$ is an inner coding of $\theta$ then the partition ${\mathcal {P}}_{\beta }$ associated to $\beta$ satisfies
  $$\begin{aligned}\forall m \, \forall A\in {{\mathcal {P}}}_\beta \, \exists B\in {{\mathcal {P}}}_\beta \text{ such } \text{ that } \theta _m(A)\subset B.\end{aligned}$$
2. Conversely, if there is a partition ${{\mathcal {P}}}$ of ${{\mathcal {A}}}$ such that
  $$\begin{aligned} \forall m \, \forall A\in {{\mathcal {P}}}\, \exists B\in {{\mathcal {P}}} \text{ such } \text{ that } \theta _m(A)\subset B, \end{aligned}$$
  then the canonical projection $\beta :{{\mathcal {A}}}\rightarrow {{\mathcal {P}}}$ defines an inner coding $(\eta , \beta )$ of $\theta$ through $\eta _m:= \beta \theta _m\beta ^{-1}$.

#### Proof

Suppose that $(\eta ,\beta )$ is an inner coding of $\theta$. Then for all *m* we have $\eta _m\circ \beta = \beta \circ \theta _m$. This implies that for all $b\in {{\mathcal {B}}}$, $\beta \theta _m$ has the same value on all elements of $\beta ^{-1}(b)$. In other words for any $A\in {{\mathcal {P}}}_\beta$ we have that $\beta \theta _m(A)$ is a singleton, and this implies that $\theta _m(A)$ must be a subset of a member of ${{\mathcal {P}}}_\beta$.

As for the converse, if ${{\mathcal {P}}}$ is a partition with the required property then we can define ${{\mathcal {B}}}:={{\mathcal {P}}}$, the code $\beta :\text{\AA }\rightarrow {{\mathcal {B}}}$ to be the map that sends $a\in {{\mathcal {A}}}$ to the member of ${{\mathcal {P}}}$ to which it belongs and for $b\in {\mathcal {B}}$, $\eta _m(b)$ is defined to be the member of ${{\mathcal {P}}}$ which contains $\theta _m(b)$, i.e., $\eta _m:= \beta \theta _m\beta ^{-1}$. $\square$

If ${\mathcal {P}}$ satisfies (2) of Lemma 3, we call the associated inner encoding the *inner encoding defined by the partition* ${{\mathcal {P}}}$.

**Fig. 1:**
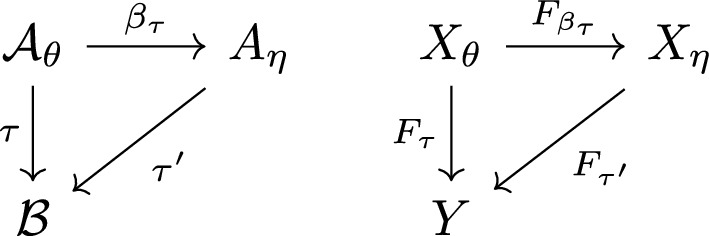
On the left: The commuting diagram of codes defined by a code $\tau$ and its inner encoding $(\eta _\tau ,\beta _\tau )$. On the right: The commutative diagram of factor maps induced by the codes on the left. Under its assumptions Theorem 4 implies that $F_{\tau '}$ is a conjugacy

Although not all codes give rise directly to inner encoded substitutions, they induce an inner encoded substitution in the following way: Let $\theta$ be a substitution on the alphabet ${{\mathcal {A}}}$ and $\tau :{{\mathcal {A}}}\rightarrow {{\mathcal {B}}}$ be a code. If ${{\mathcal {P}}}_\tau$ does not satisfy Condition (2) of Lemma 3, we can define a finer partition ${\tilde{{{\mathcal {P}}}}}_\tau$ which has this property, notably through the equivalence relation $a\sim b$ if $\forall n\ge 0,0\le m<\ell ^n$, $\tau ({\theta ^n}_m(a))=\tau ({\theta ^n}_m(b))$. We denote the associated inner encoded system by $(\eta _\tau ,\beta _\tau )$ and call it the *inner encoding defined by* $\tau$. The alphabet of $\eta _\tau$ is usually smaller than ${{\mathcal {A}}}$ and usually larger than ${{\mathcal {B}}}$. There is thus a code $\tau ':{{\mathcal {A}}}_{\eta _\tau }\rightarrow {{\mathcal {B}}}$ which comes with $\eta$ and satisfies $\tau =\tau '\circ \beta _\tau$. This is summarised in the left hand side of Fig. 1.

### Factors of substitution shifts

Let ${{\mathcal {F}}}(X)$ denote the semigroup of maps from *X* to itself. In this article we will be working extensively with the following semigroup.

#### Definition 4

The semigroup $S_\theta$ of a length-$\ell$ substitution $\theta$ over the alphabet ${{\mathcal {A}}}$ is the subsemigroup of ${\mathcal {F}}({\mathcal {A}})$ generated by the column maps $\theta _i$, $i=0,\cdots ,\ell -1$.

In this section our aim is to show that, up to conjugacy, any dynamical factor of a primitive, aperiodic, constant length substitution ${\theta }$ of trivial height is an inner encoding of a collaring $\theta ^{(n)}$ of $\theta$, where $n=(-l,r)$ with $l,r\le 1$. Later we will see using the suspension construction how the result transposes to the case of any height. As the proofs of many of the results in this section are gentle modifications of previously established results, we include them in Appendix A.

We first state in this context Theorem 4, which is a slight modification of [16, Theorem 22] when the substitution is assumed of trivial height, and which we use extensively. It focuses on shift factors which are given by a code. Such shift factors are also called $\ell$-*automatic*. While the statement of [16, Theorem 22] does not mention inner encoded substitutions, a look at its proof shows that the substitution referred to in that theorem is exactly the inner encoded substitution defined by the code. In the original statement of Theorem 4 the substitution is assumed *pair aperiodic*. This was not a restriction as there is always a power of $\theta$ which is pair aperiodic. Here we assume that $S_\theta \subset S_{\theta ^2}$, and show in Lemma 20 that this is also not a restriction as there is always a power of $\theta$ which satisfies this condition. The proof of Theorem 4, that we provide in Appendix A, is new.

#### Theorem 4

Let $\theta$ be a primitive aperiodic length-$\ell$ substitution. Suppose that $S_\theta \subset S_{\theta ^2}$. Let $F_\tau :X_\theta \rightarrow Y\subset {{\mathcal {B}}}^{\mathbb {Z}}$ be a factor which is induced by a code $\tau :{\mathcal {A}}_\theta \rightarrow {\mathcal {B}}$. Then there exists $(\eta ,\beta )$ which is inner encoded by $\theta$ and surjective code $\tau ':{\mathcal {A}}_{\eta } \rightarrow {\mathcal {B}}$ such that $\tau = \tau ' \circ \beta$ and the induced map $F_{\tau '}:X_\eta \rightarrow {{\mathcal {B}}}^{\mathbb {Z}}$ is injective and has image $Y= F_{\tau '}(X_{\eta })$. In other words $F_{\tau '}$ is a conjugacy between $X_\eta$ and *Y*.

We have summarised the statement in the second commuting diagram of Fig. 1, which follows from the first commuting diagram.

#### Proposition 5

Let $\theta$ be a primitive, aperiodic length-$\ell$ substitution, and let $F:X_\theta \rightarrow Y\subset {{\mathcal {B}}}^{\mathbb {Z}}$ be a factor map. There exists an $n=(-l,r)$-collaring $\theta ^{(n)}$ and a code $\tau :{{\mathcal {A}}}_{\theta ^{(n)}}\rightarrow {{\mathcal {B}}}$ such that $F = F_\tau \circ F_{\imath }^{-1}$. If, in addition, *Y* is an aperiodic length-$\ell$ substitution shift and *F* preserves the fixed point fibre, then $l, r \leqslant 1$.

**Fig. 2:**
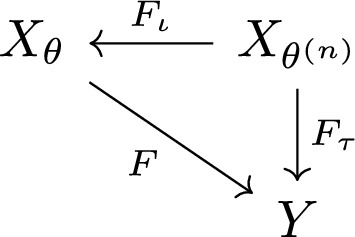
The commutative diagram for Prop. 5

The statement of Proposition 5 is summarised in Fig. 2. Its proof is based on that of [4, Proposition 3.19]; we provide it in Appendix A.

#### Corollary 2

Let $\theta$ be an aperiodic, primitive, length-$\ell$ substitution on ${\mathcal {A}}_{\theta }$.

1. If $X_\theta$ has a proper almost automorphic factor then it has a shift factor which is an almost one-to-one extension of the maximal equicontinuous factor of $X_\theta$.
2. If $F:X_\theta \rightarrow Y\subset {{\mathcal {B}}}^{\mathbb {Z}}$ is a shift factor then there exists an $n=(-l,r)$-collaring $\theta ^{(n)}$ with $l,r \le 1$, a natural number $p\ge 1$, a length-$\ell ^p$ substitution $\eta$ and a code $\beta :{{\mathcal {A}}}_{{\theta ^{(n)}}^p}\rightarrow {{\mathcal {A}}}_\eta$ such that $(\eta ,\beta )$ is an inner encoding of ${\theta ^{(n)}}^p$ and $(X_\eta , \sigma )$ is conjugate to $(Y,\sigma )$. Moreover, the conjugacy is given by a code $\tau ':{{\mathcal {A}}}_\eta \rightarrow {{\mathcal {B}}}$.

#### Proof

If $X_\theta$ has a proper almost automorphic factor then we may assume, by Theorem 20, that this factor is a proper almost automorphic extension of the maximal equicontinuous factor of $X_\theta$. By [7, Theorem 6.4], this factor is a shift factor.

As for the second statement, we apply Proposition 5 to obtain a code with its factor map $F_\tau :X_{\theta ^{(n)}}\rightarrow Y$ for some collaring $\theta ^{(n)}$ of $\theta$ such that the diagram in Fig. 2 commutes. Let *p* be such that $S_{\theta ^p}\subset S_{\theta ^{2p}}$. We apply Theorem 4 to $F_\tau :X_{{\theta ^{(n)}}^p}\rightarrow Y$ to obtain the inner encoding $(\eta _\tau ,\beta _\tau )$ of ${\theta ^{(n)}}^p$. This situation is summarised in the right half of the following diagram and its left half follows from Lemma 1 as $X_{{\theta ^{(n)}}^p }$ is equal to $X_{{\theta ^{(n)}}}$. 
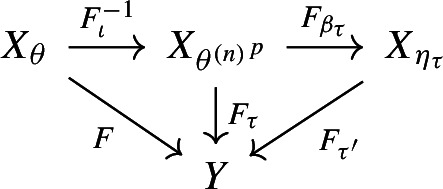
 If $n=(-l,r)$ with $l,r\le 1$ then we are done, the factor $X_\theta \rightarrow Y$ is conjugate to the factor $F_{\beta _\tau }:X_{{\theta ^{(n)}}^p}\rightarrow X_{\eta _\tau }$ where $(\eta _\tau ,\beta _\tau )$ is an inner encoding.

If $n=(-l,r)$ with $l>1$ or $r >1$ then we need one more step. As $F_\imath$ and $F_{\beta _\tau }$ are both obtained from inner encodings, they preserve the fixed point fibres. It follows that the composition ${{\tilde{F}}}:= F_{\beta _\tau }\circ F_\imath ^{-1}:X_\theta \rightarrow X_{\eta _\tau }$ preserves the fixed point fibre. We repeat the whole argument above but with $X_{\eta _\tau }$ in place of *Y*. We can apply Proposition 5, to obtain the commutative diagram 
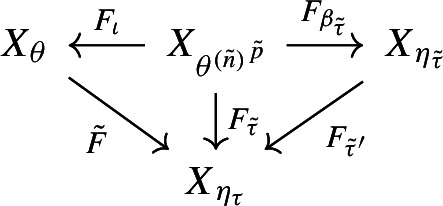
 however this time with ${{\tilde{n}}} = ({{\tilde{l}}},{{\tilde{r}}})$ and with ${{\tilde{l}}},{{\tilde{r}}}\le 1$, as $X_{\eta _\tau }$ is a substitution shift. This gives us a chain of conjugacies, namely between the factor $F:X_\theta \rightarrow Y$ and ${{\tilde{F}}}:X_\theta \rightarrow X_{\eta _\tau }$ as we saw above, and then between ${{\tilde{F}}}:X_\theta \rightarrow X_{\eta _\tau }$ and $F_{\beta _{{\tilde{\tau }}}}:X_{{\theta ^{({{\tilde{n}}})}}^{{{\tilde{p}}}}}\rightarrow X_{\eta _{{\tilde{\tau }}}}$.

$$\square $$

## Almost automorphic factors

In this section we completely characterise when an aperiodic primitive substitution $\theta$ of constant length has a proper almost automorphic factor. Corollary 2 tells us that the existence of such a factor implies that there is an inner encoding of a power of a collared version of $\theta$ whose associated substitution shift is an almost one-to-one extension of the maximal equicontinuous factor of $X_\theta$. Here we attack the converse. We will find that there are always inner encodings of a collared version of $\theta$ which have a coincidence, but the desired almost automorphic factor will exist only if the relevant inner encoding is aperiodic. We first restrict to the case of trivial height, leading to Theorem 9, and then treat the general case using the pure base of $\theta$ in Corollary 4.

### The semigroup of a length-$\ell$ substitution

We defined the semigroup $S_\theta$ of a substitution of constant length $\theta$ to be the semigroup generated by the column maps, see Definition 4. In Appendix A we provide statements and proofs of some classical results from semigroup theory. We denote by $S_\theta ^{(n)}$ the subset of maps of $S_\theta$ which have rank (size of the image) smaller or equal to *n*. If $S_\theta ^{(n)}$ is not empty then it is a two-sided ideal of *S*. Recall from (2) that the column rank of $\theta$ is the smallest rank of a product of column maps. From Corollary 8 of the appendix we obtain the following.

#### Lemma 4

Let $\theta$ be a constant length substitution. The kernel of $S_\theta$ is $S_\theta ^{(c)}$, where *c* is the column rank of $\theta$.

#### Definition 5

The *minimal sets* of the substitution $\theta$ are the images of the maps of $S_\theta$ of minimal rank. We denote the family of minimal sets by $U_\theta$, i.e.,

$$\begin{aligned} U_\theta := \{\textrm{im}f:f\in \ker S_\theta \}. \end{aligned}$$

If $U_\theta$ is a cover of ${{\mathcal {A}}}$, that is, ${{\mathcal {A}}}= \bigcup _{f\in \ker S_\theta }\textrm{im}f$, then we call the substitution *essentially surjective*. Given $U_\theta$ we define a relation on the members by $A\sim B$ if $A\cap B\ne \emptyset$. The transitive closure of this relation is an equivalence relation on $\bigcup _{f\in \ker S_\theta }\textrm{im}f$ which defines a partition which we call the *coincidence partition* and denote by ${{\mathcal {P}}}_\theta$.

#### Example 1

Consider the following substitution of length 4:

$$\begin{aligned} \theta : a \mapsto abcc \qquad b \mapsto badd \qquad c \mapsto cacd \qquad d \mapsto dbdc . \end{aligned}$$

We see that $\theta _0={{\textbf{1}}}$, the identity map. One easily checks that any product of these maps which contains at least one $\theta _i$ with $0<i<4$ has rank 2 and hence the column rank is 2 and $S_\theta = \{{{\textbf{1}}}\} \cup S_\theta ^{(2)}$. Also, $S_\theta ^{(2)}$ is the kernel. It contains the following elements:

$$\begin{aligned}&\theta _1\theta _2&: \, a \mapsto a \qquad b \mapsto b \qquad c \mapsto a \qquad d \mapsto b \\&\theta _1\theta _1&: \, a \mapsto a \qquad b \mapsto b \qquad c \mapsto b \qquad d \mapsto a \\&\theta _2&: \, a \mapsto c \qquad b \mapsto d \qquad c \mapsto c \qquad d \mapsto d \\&\theta _3 \theta _3&: \, a \mapsto d \qquad b \mapsto c \qquad c \mapsto c \qquad d \mapsto d \\&\theta _1&: \, a \mapsto b \qquad b \mapsto a \qquad c \mapsto a \qquad d \mapsto b \\&\theta _1 \theta _1\theta _2&: \, a \mapsto b \qquad b \mapsto a \qquad c \mapsto b \qquad d \mapsto a \\&\theta _3&: \, a \mapsto c \qquad b \mapsto d \qquad c \mapsto d \qquad d \mapsto c \\&\theta _3 \theta _2&: \, a \mapsto d \qquad b \mapsto c \qquad c \mapsto d \qquad d \mapsto c \end{aligned}$$

$S_\theta ^{(2)}$ has two right ideals, namely one generated by $\theta _1$ and the other by $\theta _3$. The elements of the right ideal generated by $\theta _1$ have image $\{a,b\}$ while those of the right ideal generated by $\theta _3$ have image $\{c,d\}$. Thus $U_\theta =\{\{ a,b\},\{ c,d\}\}$ and since this is a partition we have ${\mathcal {P}}_\theta =U_\theta$. For later use we also note that $S_\theta ^{(2)}$ has two left ideals, one generated by $\theta _2$ and the other by $\theta _3$.

#### Lemma 5

A primitive substitution is essentially surjective.

#### Proof

Suppose that $\theta$ is primitive. Let $a\in {{\mathcal {A}}}$ and $b\in \textrm{im}f$ for some $f\in \ker S_\theta$. By primitivity *a* occurs in $\theta ^N(b)$ for some *N*. Hence $a \in \textrm{im}g f$ for some $g\in S_\theta$. Clearly $gf\in \ker S_\theta$. Hence $a\in \bigcup _{f\in \ker S_\theta } \textrm{im}f$. $\square$

The condition of primitivity is sufficient but not necessary: the length 1 substitution $\theta =\textrm{id}$ on any alphabet ${{\mathcal {A}}}$ is not primitive, but $U_\theta =\{{{\mathcal {A}}}\}$ and therefore $\theta$ is essentially surjective. Not all substitutions are essentially surjective: the length 2 substitution on $\{a,b\}$, $\theta (a) = aa$, $\theta (b) = ab$ is not primitive and *b* does not belong to a minimal set.

#### Lemma 6

Let $\theta$ be an essentially surjective substitution. Then ${{\mathcal {P}}}_\theta$ is a partition of ${{\mathcal {A}}}$ which satisfies Condition (2) of Lemma 3. Its associated inner encoding $(\eta _{{{\mathcal {P}}}_\theta },\beta _{{{\mathcal {P}}}_\theta })$ has column rank $c=1$.

#### Proof

Let $A\in U_\theta$, that is, $A=\textrm{im}g$ for some $g\in \ker S_\theta$. Let $f\in S_\theta$. Then $f(A)\in U_\theta$ as $fg\in \ker S_\theta$. Now if $A\cap A'\ne \emptyset$ then also $f(A)\cap f(A')\ne \emptyset$. Thus if two members $A,A'$ of the cover $U_\theta$ belong to the same member of ${\mathcal {P}}_\theta$ then also *f*(*A*) and $f(A')$ belong to the same member of ${\mathcal {P}}_\theta$. This implies Condition (2) of Lemma 3 for ${\mathcal {P}}_\theta$. Furthermore, if *f* belongs to the kernel of $S_\theta$ then $f(A)=\textrm{im}fg = \textrm{im}f$ and so *f*(*A*) is the same for all $A\in U_\theta$. Hence $\beta _{{{\mathcal {P}}}_\theta } f \beta _{{{\mathcal {P}}}_\theta }^{-1}$ has rank 1, and so $\eta _{{{\mathcal {P}}}_\theta }$ has column rank 1. $\square$

#### Definition 6

Let $\theta$ be an essentially surjective substitution with coincidence partition ${{\mathcal {P}}}_\theta$. We call the inner encoding defined by ${{\mathcal {P}}}_\theta$ the *canonical inner encoding of* $\theta$. We denote this inner encoding $(\eta _{{{\mathcal {P}}}_\theta },\beta _{{{\mathcal {P}}}_\theta })$ by $(\eta _{\theta },\beta _{\theta })$.

The next lemma tells in particular us how the canonical inner encodings of $\theta$ and its powers are related.

#### Lemma 7

Let $\theta$ be a substitution and $N\ge 1$. Then $\ker S_{\theta ^N}=\ker S_\theta$. In particular $U_{\theta }$ and $U_{\theta ^N}$ coincide. Furthermore, if the substitution is essentially surjective then the inner encodings $(\eta _{\theta ^N},\beta _{\theta ^N})$ and $(\eta _\theta ,\beta _\theta )$ associated to $\theta ^N$ and $\theta$ satisfy $\eta _{\theta ^N} = {\eta _\theta }^N$ and $\beta _{\theta ^N}=\beta _{\theta }$.

#### Proof

By definition of the column rank, $\theta$ and $\theta ^N$ have the same column rank. Clearly $S_{\theta ^N}^{(c)}\subset S_\theta ^{(c)}$. To see that the inclusion is surjective, recall that any element *f* of $\ker S_\theta$ is completely regular and hence we can factorise $f = f {f^0}^{N-1}$, where we recall that $f^0$ is the idempotent generated by *f*. This shows that $\ker S_{\theta }=\ker S_{\theta ^N}$ and immediately implies $U_{\theta }=U_{\theta ^N}$ and, if $U_{\theta }$ is a cover, that $\beta _{\theta }=\beta _{\theta ^N}$. Let $0\le j\le \ell ^N-1$. There are $j_1,...,j_N$ such that ${\theta ^N}_j = \theta _{j_1}\cdots \theta _{j_N}$. Hence ${\eta _{\theta ^N}}_j = \beta _\theta \theta _{j_1}\cdots \theta _{j_N} \beta _\theta ^{-1} = {\eta _\theta }_{j_1}\cdots {\eta _\theta }_{j_N}$. $\square$

As $\eta _\theta$ has column rank $c=1$ and *h* divides *c*, it has a coincidence and trivial height. Its associated dynamical system is thus almost automorphic. However, it need not be aperiodic, nor, if it is aperiodic, does it have to have the same maximal equicontinuous factor as $\theta$. The latter happens always if $\theta$ has non-trivial height, because then the maximal equicontinuous factor of $\eta _\theta$ is strictly smaller than that of $\theta$. In the rest of this section we will show two things: under the assumption of trivial height, if $\eta _{\theta ^{(n)}}$ is periodic for all the collared versions of $\theta$ (we only need $n=(-l,r)$ with $l,r\le 1$) then $X_\theta$ does not admit a proper almost automorphic factor, and if the height of $\theta$ is non-trivial we can reduce the task to working with the pure base of $\theta$.

We provide an algebraic property of $S_\theta$ characterising column rank 1. A semigroup is *left zero* if every element acts as a zero element when multiplying from the left, i.e. $xy=x$ for all $x,y\in S$. If *S* is completely simple, then it is left zero if and only if the ${{\mathcal {R}}}$-relation is trivial (equal to the diagonal relation).

#### Lemma 8

A constant length substitution $\theta$ has column rank 1 if and only if $\ker S_\theta$ is a left zero semigroup. If this is the case and if $\theta$ is essentially surjective then $\ker S_\theta \ni x\mapsto \textrm{im}x \in {{\mathcal {A}}}$ is a bijection.

#### Proof

$\theta$ has column rank 1 if and only if $S_\theta$ contains an element of rank 1 which is equivalent to saying that $\ker S_\theta$ contains exactly the maps of rank 1 of $S_\theta$. Any collection of rank 1 maps from ${{\mathcal {F}}}({{\mathcal {A}}})$ forms a left zero semigroup.

Now, suppose that the column rank is $c>1$. If $S_\theta$ has more than one minimal left ideal, then $\ker S_\theta$ is not a left zero semigroup. If $S_\theta$ has a unique minimal left ideal, then, as we will see in Theorem 13, $\theta$ admits an inner encoding $\eta$ such that $S_\eta$ is a non-trivial group. In particular $\ker S_\eta = S_\eta$ and since an inner encoding induces an epimorphism from $\ker S_\theta$ to $\ker S_\eta$, $\ker S_\theta$ cannot be left zero.

Any rank 1 map can be identified with the unique element in its image. Hence $\ker S_\eta \ni x\mapsto \textrm{im}x \in {{\mathcal {A}}}$ is injective and, if $U_\theta$ is a cover, also surjective. $\square$

### The canonical outer encoding

#### Definition 7

Let $\theta$ and $\eta$ be length-$\ell$ substitutions. We say that $\eta$ is *outer encoded* by $\theta$ if there is an epimorphism $\Phi :S_\theta \rightarrow S_\eta$ such that $\eta _m= \Phi (\theta _m)$ for $0\le m \le \ell -1$.

We may also say that $(\eta ,\Phi )$ is outer encoded by $\theta$, that $\eta$ is an outer encoded substitution of $\theta$, or that $\eta$ is an outer encoding of $\theta$. Note that if $(\eta ,\beta )$ is an inner encoding of $\theta$, then $\eta$ is outer encoded with epimorphism

$$\begin{aligned} \Phi _\beta (f) = \beta f \beta ^{-1}. \end{aligned}$$

However an outer encoding does not necessarily define an inner encoding or induce a factor map; see Example 4.

#### Corollary 3

If $(\eta ,\Phi )$ is outer encoded by $\theta$, then the restriction of $\Phi$ to $\ker S_\theta$ is an epimorphism onto $\ker S_\eta$.

#### Proof

This follows from Lemma 24 as $S_\theta$ is finite and hence admits a kernel. $\square$

#### Definition 8

Let $\theta$ be a length-$\ell$ substitution with column rank *c*. Let ${{\mathcal {B}}}= \ker (S_\theta )/{{\mathcal {R}}}$, and $^c\Phi :S_\theta \rightarrow {\mathcal {F}}({\mathcal {B}})$ be the morphism defined by

4 $$\begin{aligned}  ^c\Phi (f)( [x]_{{\mathcal {R}}}):= [fx]_{{\mathcal {R}}}. \end{aligned}$$

The *canonical outer encoding of* $\theta$ is the substitution $^c\theta : {{\mathcal {B}}}\rightarrow {{\mathcal {B}}}^{\ell }$ defined by

5 $$\begin{aligned}  ^c\theta _m :=  ^c\Phi (\theta _m). \end{aligned}$$

The semigroup of a bijective substitution is a group. Therefore $S_\theta = \ker S_\theta$ and $S_\theta /{{\mathcal {R}}}$ consists of a single point. Hence the canonical outer encoding of $\theta$ is the one letter periodic substitution, which is, of course, a trivial inner encoding.

#### Example 2

The canonical outer encoded substitution of the collared Thue-Morse system $\theta ^{(0,1)}$ is the period doubling substitution, and it is also an inner encoding, see Sect. 3.5. Note that we must take a collared version of $\theta$, as the canonical outer encoding of any bijective substitution is trivial.

We are interested in finding almost automorphic factors. The following lemma explains our interest in canonical outer encodings. Note there is no need to assume that $\theta$ is primitive.

#### Lemma 9

The canonical outer encoding $(  ^c\theta ,  ^c\Phi )$ of $\theta$ is primitive and has column rank 1, i.e., has trivial height and a coincidence.

#### Proof

We will show that there is an element $f\in S_{ ^c\theta }$ whose image contains only one letter; this implies that $^c\theta$ has column rank 1 and therefore a coincidence and trivial height. By Lemma 24, $^c\Phi$ restricts to an epimorphism from $\ker S_\theta$ to $\ker S_{ ^c\theta }$. Given $f\in \ker S_\theta$ and $[y]_{{\mathcal {R}}}\in \ker S_\theta /{{\mathcal {R}}}$ we have $^c\Phi (f)([y]_{{\mathcal {R}}}) = [fy]_{{\mathcal {R}}}= [f]_{{\mathcal {R}}}$. We thus see that the image of $^c\Phi (f)$ contains only the letter $[f]_{{\mathcal {R}}}$. Hence the column rank is 1.

To see that $^c\theta$ is primitive, we need to show that for any two letters $[x],[y]\in \ker S_{ ^c\theta }/{{\mathcal {R}}}$, there is an $f\in S_{ ^c\theta }$ such that $[fx]=[y]$. As any left ideal of $\ker S_\theta$ intersects any right ideal of $\ker S_\theta$, any two classes $[x],[y]\in \ker S_{ ^c\theta }/{{\mathcal {R}}}$ have representatives *x*, *y* which belong to the same ${{\mathcal {L}}}$-class. This means that there is $f\in S_{ ^c\theta }$ such that $y=fx$. $\square$

#### Proposition 6

Let $\theta$ be an essentially surjective substitution and $( ^c\theta , ^c\Phi )$ be the canonical outer encoding of $\theta$. Then $^c\theta$ is an inner encoding of $\theta$ if and only if $U_\theta$ is a partition of ${{\mathcal {A}}}$. In this case, and upon identifying $\ker S_\theta /{{\mathcal {R}}}$ with $U_\theta$, we have $^c\theta = \eta _\theta$, the canonical inner encoding of $\theta$.

#### Proof

Recall that the alphabet of $^c\theta$ is $\ker S_\theta /{{\mathcal {R}}}$. By Lemma 23 the map $\ker S_\theta /{{\mathcal {R}}}\ni [x]_{{\mathcal {R}}}\mapsto \textrm{im}x \in U_\theta$ is a bijection.

If $U_\theta$ is a partition of ${{\mathcal {A}}}$ then it coincides with the coincidence partition ${{\mathcal {P}}}_\theta$ and hence we may identify $\beta _\theta :{{\mathcal {A}}}\rightarrow {{\mathcal {P}}}_\theta$ with the code $a \mapsto [x]_{{\mathcal {R}}}$ where *x* is any function from $\ker S_\theta$ which contains *a* in its image. Say $a=x(b)$. We then have $^c\theta _m(\beta _\theta (a)) = [\theta _m x]_{{\mathcal {R}}}$ while $\beta _\theta (\theta _m(a)) = \beta _\theta (\theta _mx(b)) = [\theta _m x]_{{\mathcal {R}}}$. Hence $^c\theta = \eta _\theta$.

Suppose that $^c\theta$ is an inner encoding of $\theta$, i.e. there is a code $\beta :{{\mathcal {A}}}\rightarrow \ker S_\theta /{{\mathcal {R}}}$ such that $\beta \theta _m = ^c\theta _m\beta$. By Lemma 9, $^c\theta$ has column rank 1 and so $\ker S_{ ^c\theta }$ contains only rank 1 maps. Hence, for $g_1, g_2\in \ker S_{ ^c\theta }$ the condition $g_1\ne g_2$ is equivalent to $\textrm{im}g_1\cap \textrm{im}g_2=\emptyset$. Let $f_i\in ( ^c\Phi )^{-1}(g_i)\cap \ker S_\theta$. If $\textrm{im}f_1\cap \textrm{im}f_2\ne \emptyset$ then there are $a_1,a_2\in {{\mathcal {A}}}$ such that $f_1(a_1)= f_2(a_2)$. It follows that $g_1(\beta (a_1))=\beta f(a_1) =\beta f(a_2) =g_2(\beta (a_2))$, hence $\textrm{im}g_1\cap \textrm{im}g_2\ne \emptyset$, hence $g_1= g_2$. Thus $\textrm{im}f_1\cap \textrm{im}f_2\ne \emptyset$ implies $^c\Phi (f_1) =  ^c\Phi (f_2)$ which means that $f_1$ and $f_2$ are ${{\mathcal {R}}}$-related. By Lemma 22 they then have the same image. Thus the elements of $U_\theta$ either coincide or do not intersect. As we assumed that $U_{{\mathcal {A}}}$ covers ${{\mathcal {A}}}$ it is a partition of ${{\mathcal {A}}}$. $\square$

#### Example 3

We return to Example 1 whose cover of minimal sets $U_\theta$ is a partition and thus the canonical outer encoding is inner encoded. Setting $A=\{a,b\}$ and $C=\{c,d\}$ the inner encoded substitution is given by

$$\begin{aligned} \eta _\theta : A&\mapsto AACC \hspace{3em} C \mapsto CACC . \end{aligned}$$

Clearly it is aperiodic, and hence $X_{\eta _\theta }$ is a proper, almost automorphic factor of $X_\theta$.

#### Example 4

Consider the substitution

$$\begin{aligned} \theta : a&\mapsto acaef \hspace{3em} b \mapsto bdbde \hspace{3em} c \mapsto ceccg \\ d&\mapsto dfbde \hspace{3em} e \mapsto egaef \hspace{3em} f \mapsto dfbfg \hspace{3em} g \mapsto cecge. \end{aligned}$$

Its minimal sets are $U_\theta =\{ A=\{a,b,c \}, B=\{c,d,e \},C=\{e,f,g \} \}$. The canonical outer encoding is $A \mapsto ABABC, B \mapsto BCABC, C \mapsto BCACC.$ The cover $U_\theta$ does not form a partition. It generates the partition ${\mathcal {P}}_\theta = \{ \{a,b,c,d,e,f,g\} \}$ which leads to the periodic substitution

$$\begin{aligned} \eta _\theta : D&\mapsto DDDDD \end{aligned}$$

and thus we cannot conclude that $X_\theta$ has a proper almost automorphic factor.

#### Lemma 10

Any inner encoding of an essentially surjective substitution is essentially surjective.

#### Proof

Let $(\eta ,\beta )$ be inner encoded by $\theta$, $\beta :{{\mathcal {A}}}\rightarrow {{\mathcal {B}}}$. Let $b\in {{\mathcal {B}}}$. As $U_\theta$ covers ${{\mathcal {A}}}$ there is $a\in {{\mathcal {A}}}$ and $f\in \ker S_\theta$ such that $\beta (f(a))=b$. Hence $\beta f \beta ^{-1}(\beta (a))=b$. Moreover, $\beta f \beta ^{-1}\in \ker S_\eta$. Hence $b\in U_\eta$. $\square$

#### Proposition 7

Let $\theta$ be a substitution which is essentially surjective. Let $^c\theta$ be the canonical outer encoding of $\theta$. If $(\eta ,\Phi )$ is outer encoded by $\theta$, and $\eta$ is essentially surjective and has column rank 1, then $(\eta ,\Phi )$ is outer encoded by $^c\theta$.

#### Proof

Let $( ^c\eta ,  ^c\Phi _\eta )$ be the canonical outer encoding of $\eta$. We claim that there is a morphism $\varphi :S_{ ^c\theta } \rightarrow S_{ ^c\eta }$ such that the diagram 
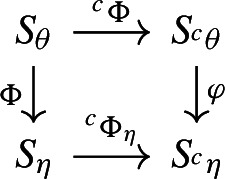


is commutative. By Lemma 24$\Phi (\ker S_\theta )=\ker S_\eta$ and $\Phi$ preserves the ${{\mathcal {R}}}$-relation. Therefore, given $t,s\in S_\theta$ and $x\in \ker S_\theta$, $[tx]_{{{\mathcal {R}}}_\theta }=[sx]_{{{\mathcal {R}}}_\theta }$ implies $[\Phi (t) \Phi (x)]_{{{\mathcal {R}}}_\eta }=[\Phi (s) \Phi (x)]_{{{\mathcal {R}}}_\eta }$ (here ${{\mathcal {R}}}_\theta$ is the ${{\mathcal {R}}}$-relation on $S_\theta$ and ${{\mathcal {R}}}_\eta$ is the ${{\mathcal {R}}}$-relation on $S_\eta$). Also by Lemma 24, any $y\in \ker S_\eta$ has a pre-image under $\Phi$ in $\ker S_\theta$, so we see that $^c\Phi (t)= ^c\Phi (s)$, which is $[tx]_{{{\mathcal {R}}}_\theta }=[sx]_{{{\mathcal {R}}}_\theta }$ for all $x\in \ker S_\theta$, implies $^c\Phi _\eta (\Phi (t))= ^c\Phi _\eta (\Phi (s))$, which is $[\Phi (t) y]_{{{\mathcal {R}}}_\eta }=[\Phi (s) y]_{{{\mathcal {R}}}_\eta }$ for all $y\in S_\eta$. Thus $\varphi$ is well defined through the formula $\varphi ( ^c\Phi (t)) =  ^c\Phi _\eta (\Phi (t))$.

As $\eta$ has column rank 1 and is essentially surjective, $\ker S_\eta$ can be identified with the alphabet of $\eta$ and therefore $^c\Phi _\eta :S_\eta \rightarrow {{\mathcal {F}}}(\ker S_\eta )$ is injective. Hence $\Phi (t)=(^c\Phi _\eta )^{-1}(\varphi ( ^c\Phi (t)))$, showing that it factors through $^c\Phi$. $\square$

#### Definition 9

Let $\theta$ be an essentially surjective substitution. Its *maximal inner encoding with column rank *1 is an inner encoded substitution $\eta$ of $\theta$, such that any other inner encoding of $\theta$ which has column rank 1 factors, via an inner encoding, through $\eta$.

We now show that maximal inner encodings with column rank 1 exist.

#### Theorem 8

Let $\theta$ be an essentially surjective substitution. Its maximal inner encoding with column rank 1 is the canonical inner encoding $\eta _\theta$ of $\theta$. In particular, $\theta$ admits an aperiodic inner encoding with column rank 1 if and only if its canonical inner encoding $\eta _\theta$ is aperiodic.

#### Proof

By Lemma 6, $\eta _\theta$ is an inner encoding with column rank 1. Suppose that $\eta$ is an inner encoding of $\theta$ with column rank 1. We saw in Proposition 7 that it must be an outer encoding of the canonical outer encoding $^c\theta$. Furthermore the epimorphism $\Phi :S_{ ^c\theta }\rightarrow S_\eta$ restricts to an epimorphism from $\ker S_{ ^c\theta }$ to $\ker S_{\eta }$. Since both have column rank 1, this restriction of $\Phi$ is an epimorphism between left zero semigroups, hence a surjective map from the alphabet of $^c\theta$, which is $U_\theta$, to the alphabet of $\eta$. On the other hand, the code from ${{\mathcal {A}}}$ to the alphabet of $\eta$ is given by a partition ${{\mathcal {P}}}$. Any member of $U_\theta$ must therefore be a subset of an element of ${{\mathcal {P}}}$. Thus any element of ${{\mathcal {P}}}_\theta$ is a subset of an element of ${{\mathcal {P}}}$. It follows that $\eta$ is an inner encoding of the inner encoding defined by ${{\mathcal {P}}}_\theta$, which is the canonical inner encoding $\eta _\theta$. $\square$

### Trivial height

Recall that a substitution has a coincidence if and only if its height coincides with its column rank. In this subsection we consider the case in which $\theta$ has height 1. This allows us to exploit the above results about inner encoded substitutions with column rank 1 for the analysis of almost automorphic factors.

#### Theorem 9

Let $\theta$ be a primitive substitution of trivial height. Then $X_\theta$ has a proper almost automorphic factor if and only if the canonical inner encoding associated to $\theta ^{(n)}$, for some $n=(-l,r)$, $0\le l,r\le 1$, is aperiodic.

We remark that it is sufficient to check whether the canonical inner encoding of $\theta ^{(-1,1)}$ is aperiodic, as all other $\theta ^{(n)}$ are inner encodings of $\theta ^{(-1,1)}$. However if one suspects that $X_\theta$ has a proper almost automorphic factor, in practice it is easier to check whether $X_\theta$ or $X_{\theta ^{(0,1)}}$ give almost automorphic factors, especially if doing these computations by hand.

#### Proof

Suppose that $X_\theta$ has a proper almost automorphic factor. By Cor. 2, we may assume that this factor is a shift factor which is an almost one-to-one extension of the maximal equicontinuous factor of $X_\theta$, and furthermore, that it is given by some inner encoding of ${\theta ^{(n)}}^p$ for some $|n|\le 1$ and $p\ge 1$. As the factor is properly almost automorphic, this inner encoding must be aperiodic and have column rank *h*, the height of $\theta$. But by assumption, $h=1$. It now follows from Theorem 8 that the canonical inner encoding $\eta _{(\theta ^{(n)})^{p}}$ is aperiodic and has column rank 1. By Lemma 7$\eta _{{\theta ^{(n)}}^{p}}={\eta _{\theta ^{(n)}}}^{p}$ and so also $\eta _{\theta ^{(n)}}$ is aperiodic and has column rank 1.

Conversely, the substitution shift defined by the canonical inner encoding of a collaring $\theta ^{(n)}$ with $|n|\le 1$ is an almost automorphic factor of $X_\theta$, as canonical inner encodings have column rank 1. This factor is equicontinuous if and only if the substitution is periodic.

$$\square $$

#### Example 5

Consider the Rudin-Shapiro substitution, which is defined as

$$\begin{aligned} \theta : a&\mapsto ac \hspace{3em} b \mapsto dc \hspace{3em} c \mapsto ab \hspace{3em} d \mapsto db . \end{aligned}$$

It is known that this substitution has a proper almost automorphic factor, see for example [1]; we redo the calculation in our setting. Its minimal sets are $\{\{a,d\},\{b,c\} \}$, which form a partition but which yield a periodic substitution. The substitution $\theta ^{(0,1)}$ is

$$\begin{aligned} \theta ^{(0,1)}: A&\mapsto BF \hspace{3em} B \mapsto BE \hspace{3em} C \mapsto HE \hspace{3em} D \mapsto HF \\ E&\mapsto AC \hspace{3em} F \mapsto AD \hspace{3em} G \mapsto GD \hspace{3em} H \mapsto GC, \end{aligned}$$

and the minimal sets are $\{\{A,H\},\{B,G\},\{C,F\},\{E,D\} \}= \{ p,q,r,s\}$, which again form a partition, and whose associated substitution is

$$\begin{aligned} \eta _{\theta ^{(0,1)}}: p&\mapsto qr \hspace{3em} q \mapsto qs \hspace{3em} r \mapsto ps \hspace{3em} s \mapsto pr, \end{aligned}$$

which can be verified to be aperiodic.

#### Example 6

We return to Example 4 which has height 1. We saw that the coincidence partition of $\theta$ contains only one element and so $\theta$ has no aperiodic inner encoding with a coincidence. By the comment after Theorem 9, it is enough to check whether the canonical inner encoding of $\theta ^{(-1,1)}$ is aperiodic. A more elaborate calculation shows that the coincidence partition of $\theta ^{(-1,1)}$ has three elements ${\mathcal {P}}_{\theta ^{(-1,1)} }=\{\alpha ,\beta ,\gamma \}$ and that the canonical inner encoded substitution is given by

$$\begin{aligned} \eta _{\theta ^{(-1,1)}}: \alpha&\mapsto \beta \gamma \beta \alpha \alpha \hspace{3em} \beta \mapsto \beta \gamma \beta \alpha \alpha \hspace{3em} \gamma \mapsto \beta \gamma \beta \alpha \alpha , \end{aligned}$$

which is periodic. We conclude that $X_\theta$ does not admit a proper almost automorphic factor.

### Non-trivial height

One direction of Theorem 9 does not need the assumption of trivial height. Indeed, if the canonical inner encoding $\eta _\theta$ of $\theta$ is aperiodic then $X_{\eta _\theta }$ is a proper almost automorphic factor of $X_\theta$, though one with trivial height. Using Theorem 20 we can then even obtain a proper almost automorphic factor which has the same maximal equicontinuous factor as $X_\theta$. But, as will show in the next example, if the height is not trivial then the existence of a proper almost automorphic factor does not imply that it is given by an inner encoding of $\theta ^{(-1,1)}$.

#### Example 7

The substitution

$$\begin{aligned} \theta : a&\mapsto aba \qquad b \mapsto bac \qquad c \mapsto cab \end{aligned}$$

has height 2 and column rank 2. So $X_\theta$ is almost automorphic. It thus has a proper almost automorphic factor (namely itself). The cover of minimal sets is given by $U_\theta = \{\{a,b\},\{a,c\}\}$ and so we see that the concidence partition has only one element and the canonical inner encoding is periodic. The collared substitution

$$\begin{aligned} \theta ^{(-1,1)} :&A \mapsto ADB \qquad B \mapsto BEA\qquad C \mapsto EAC\qquad D \mapsto EAD\qquad E \mapsto CAC \end{aligned}$$

has minimal sets

$$\begin{aligned} U_{\theta ^{(-1,1)}} = \{\{A,C\},\{A,D\},\{A,E\},\{B,C\},\{B,D\},\{B,E\}\}. \end{aligned}$$

Again the partition generated by $U_{\theta ^{(-1,1)}}$ contains only one element so that the canonical inner encoding is periodic. This shows that the condition of trivial height in Theorem 9 cannot be dropped.

To overcome the problem outlined in the last example we will work with the pure base of $\theta$. We recall from [5] the following results: If $\theta$ is primitive and has height *h*, then there exists a $\sigma ^h$-periodic clopen partition of $X_\theta$, $X_\theta = \bigsqcup _{k\in {\mathbb {Z}}/h{\mathbb {Z}}} X_\theta ^k$, and $\sigma (X_\theta ^k) = X_\theta ^{k+1}$. Moreover, $X_\theta ^0$ is invariant under $\theta$ and there exists a subset ${{\mathcal {A}}}'\subset {{\mathcal {A}}}^h$ such that $X_\theta ^0$ consists precisely of the sequences $x\in X_\theta$ for which $x_0\dots x_{h-1}\in {{\mathcal {A}}}'$. Define a substitution $\theta '$ on ${{\mathcal {A}}}'$ as follows: Given $a_0\dots a_{h-1}\in {{\mathcal {A}}}'$ write $\theta (a_0\dots a_{h-1})=a'_0\dots a'_{h\ell -1}$ and set

$$\begin{aligned} \theta '_k(a_0\dots a_{h-1}) = a'_{kh}\dots a'_{(k+1)h-1}. \end{aligned}$$

$\theta '$ is called the pure base of $\theta$. It corresponds to the restriction of $\theta$ to $X_\theta ^0$ but expressed in the alphabet ${{\mathcal {A}}}'$.

The suspension of a ${\mathbb {Z}}$-action $(X,\varphi )$ with ${\mathbb {Z}}/h{\mathbb {Z}}$ is the space $X\times {\mathbb {Z}}/h{\mathbb {Z}}$ equipped with the ${\mathbb {Z}}$-action

6 $$\begin{aligned} T_{\varphi }(x,i):= {\left\{ \begin{array}{ll} (x,i+1) &  \text { if } 0\le i<h-1 \\ (\varphi (x), 0) &  \text { if } i=h-1 \end{array}\right. } \end{aligned}$$

The shift $(X_\theta ,\sigma )$ is, for a substitution of height *h*, topologically conjugate to a suspension of the substitution shift $(X_{\theta '}, \sigma )$ with the finite group ${\mathbb {Z}}/h{\mathbb {Z}}$. Here we have denoted the shift action on $X_{\theta '}$ by $\sigma '$ in order to distinguish it from the shift action $\sigma$ on $X_\theta$. The conjugacy is given by

$$\begin{aligned} X_{\theta '}\times {\mathbb {Z}}/h{\mathbb {Z}}\ni (x',i) \mapsto \sigma ^i(x)\in X_{\theta }, \end{aligned}$$

where on the left hand side $x'$ is a sequence of letters from ${{\mathcal {A}}}'$, that is, a sequence of allowed (for $\theta$) words of length *h* whereas on the right hand side *x* is the sequence of letters from ${{\mathcal {A}}}$ that one obtains when one interprets $x'$ as a sequence of letters in ${{\mathcal {A}}}$. Note that $(x',i)\mapsto (\sigma '(x'),i)$ on the left corresponds to $\sigma ^i(x)\mapsto \sigma ^{h+i}(x)$ on the right. The suspension construction is functorial and immediately implies:

- If $F:(X_{\theta '},\sigma ')\rightarrow (Y,\varphi )$ is a factor map then $F\times {{\textbf{1}}}:(X_{\theta '}\times {\mathbb {Z}}/h{\mathbb {Z}},T_{\sigma '})\rightarrow (Y\times {\mathbb {Z}}/h{\mathbb {Z}},T_{\varphi })$ is a factor map and any factor map of $(X_{\theta '}\times {\mathbb {Z}}/h{\mathbb {Z}},T_{\sigma '})$, up to a rotation, arises in this way. In particular the MEF of $(X_\theta ,\sigma )$ is conjugate to $({\mathbb {Z}}_\ell \times {\mathbb {Z}}/h{\mathbb {Z}}, T_{+1})$.
- $(Y,\varphi )$ is almost automorphic if and only if $(Y\times {\mathbb {Z}}/h{\mathbb {Z}},T_{\varphi })$ is almost automorphic.

Recall that a topological dynamical system (*X*, *T*) is a minimal nontrivial almost automorphic extension of an odometer if and only if it is topologically conjugate to a shift [7, Theorem 6.4]. Combining this with the remarks above, we obtain the following.

#### Proposition 10

Let $\theta$ be a primitive, aperiodic substitution of constant length, with pure base $\theta '$. Then $(X_\theta , \sigma )$ has a proper almost automorphic factor if and only if $(X_{\theta '}, \sigma ')$ has a proper almost automorphic factor.

#### Corollary 4

Let $\theta$ be a primitive, aperiodic substitution of constant length, with pure base $\theta '$. Then $X_\theta$ has a proper almost automorphic factor if and only if the canonical inner encoding of $\theta '^{(-1,1)}$ is aperiodic.

We describe how to construct the desired almost automorphic factor of $X_{\theta }$ when $X_{\theta '}$ has an aperiodic almost automorphic factor $X_{\eta '}$ via the map $F':X_{{\theta '}}\rightarrow X_{\eta '}$. Note that $\eta '$ necessarily has height 1. Define a new length-$\ell$ substitution $\eta$ with alphabet ${\mathcal {A}}_\eta =\{a_j \,:\, a\in {\mathcal {A}}_{\eta '},1\le j \le h\}$ as follows. Define ${\mathfrak {i}}: {\mathcal {A}}_{\eta '} \rightarrow {\mathcal {A}}_\eta ^{h}$ by ${\mathfrak {i}}(a)= a_1 \dots a_h$. Now let $\eta$ be the unique length-$\ell$ substitution which satisfies $\eta \circ {\mathfrak {i}} = {\mathfrak {i}} \circ \eta '$. That is, we “split” each $a\in {\mathcal {A}}_{\eta '}$ into *h* different letters $a_1,\dotsc ,a_h$ in such a way that the concatenation of the length $\ell$ words $\eta (a_1)\cdots \eta (a_h)$ is the word obtained from ${\eta '}(a)$ by splitting every letter. As defined, $\eta$ has height *h* and pure base ${\eta '}$. Thus, $X_\eta$ is also a suspension of $X_{\eta '}$ over ${\mathbb {Z}}/h{\mathbb {Z}}$. It can be seen that $X_\eta$ is almost automorphic over $({\mathbb {Z}}_{{\bar{\ell }},h}, +1)$. See Example 8 for such a construction.

### Two-letter collaring of bijective substitutions and their canonical outer encodings

In this section we apply our results above to study when a bijective substitution shift has a proper almost automorphic factor. This reproduces the results of Martin [15] and those of Herning [10] in our semigroup based approach. In the process we revisit the work in [12, Section 4]. We start with bijective substitutions of trivial height. Remember that for bijective substitutions, $S_\theta$ is a group and so $^c\theta$ is trivial, being defined on a one letter alphabet. This does not mean that a bijective substitution shift does not admit a proper almost automorphic substitutional factor, but only that these may only be seen when working with the collared versions of the substitution. We consider here only the collaring $\theta ^{(0,1)}$.

For $\theta$ a bijective substitution on ${{\mathcal {A}}}$, let ${{\mathcal {A}}}^{(2)}$ be the set of allowed two-letter words for $\theta$. Recall that the 2-collared substitution $\theta ^{(0,1)}$ associated to $\theta$ is the substitution on ${{\mathcal {A}}}^{(2)}$ of the same length given by

$$\begin{aligned} \theta ^{(0,1)}_m(a,b) = (\theta _m(a),\theta _{m+1}(a)), \quad 0\le m<\ell -1, \quad \theta ^{(0,1)}_{\ell -1}(a,b) = (\theta _{\ell -1}(a), \theta _{0}(b)) \end{aligned}$$

We assume that $\theta _0=\theta _{\ell -1}={{\textbf{1}}}$ so that all $\theta$-periodic points are fixed; thus all maps $\theta ^{(0,1)}_m$ with $m<\ell -1$ have rank $c=|{{\mathcal {A}}}|$ while $\theta ^{(0,1)}_{\ell -1}$ is equal to the identity on ${{\mathcal {A}}}^{(2)}$ and hence has rank equal to $|{{\mathcal {A}}}^{(2)}|$. We know that the column rank of a substitution is a conjugacy invariant and so the column rank of $\theta ^{(0,1)}$ must also be *c*. The kernel of $S_{\theta ^{(0,1)}}$ is generated by $\theta _m \mathrm {pr_1}\times \theta _{m+1} \mathrm {pr_1}$, $m<\ell -1$, where $\mathrm {pr_1}:{{\mathcal {A}}}^{(2)}\rightarrow {{\mathcal {A}}}$ is the projection onto the first factor. Recall that the right ideals of $\ker S_{\theta ^{(0,1)}}$ are in one-to-one correspondence to the images of these maps. As $\theta _m$ is bijective, the image of $\theta _m \mathrm {pr_1}\times \theta _{m+1} \mathrm {pr_1}$ coincides with that of $\mathrm {pr_1}\times \theta _{m+1}\theta _m^{-1} \mathrm {pr_1}$ and so uniquely is determined by the map $\theta _{m+1}\theta _{m}^{-1}$. We thus see that the set of right ideals of $\ker S_{\theta ^{(0,1)}}$ is in one-to-one correspondence the set

$$\begin{aligned} I_\theta :=\{\theta _{m+1}\theta _{m}^{-1}|m=0,\cdots ,\ell -2\} \end{aligned}$$

which plays a prominent role in the description of the Ellis semigroup of the substitution shift $(X_\theta ,\sigma )$ and is also called the *R*-set of the substitution [12]. This gives us the first part of

#### Corollary 5

The alphabet of the canonical outer encoding $^c\theta ^{(0,1)}$ of $\theta ^{(0,1)}$ can be identified with $I_\theta$. Under this identification it is given by

$$\begin{aligned} { ^c\theta ^{(0,1)}}_m\bigg (\theta _i\theta _{i-1}^{-1}\bigg ) = \theta _{m+1}\theta _{m}^{-1},\quad 0\le m< \ell -1,\quad { ^c\theta ^{(0,1)}}_{\ell -1}\bigg (\theta _i\theta _{i-1}^{-1}\bigg )=\theta _i\theta _{i-1}^{-1} \end{aligned}$$

It is an aperiodic inner encoding of $\theta ^{(0,1)}$ if and only if for all $f,g\in I_\theta$ and $\forall a\in {{\mathcal {A}}}$: $f(a)\ne g(a)$. Thus the following is a necessary condition for $^c\theta ^{(0,1)}$ to be an inner encoding:

7 $$\begin{aligned} |I_\theta | \times |{{\mathcal {A}}}| = |{{\mathcal {A}}}^{(2)}| \end{aligned}$$

and so the code of the inner encoding is a $|{{\mathcal {A}}}|$-to-1 map.

#### Proof

Recall that the canonical outer encoding is an inner encoding if the images of the maps $\theta _m \mathrm {pr_1}\times \theta _{m+1} \mathrm {pr_1}$, $m<\ell -1$, either coincide or do not overlap. As the images of $\theta _m \mathrm {pr_1}\times \theta _{m+1} \mathrm {pr_1}$ and $\theta _{m'} \mathrm {pr_1}\times \theta _{m'+1} \mathrm {pr_1}$ coincide if and only if $\theta _m\theta _{m-1}^{-1} = \theta _{m'}\theta _{m'-1}^{-1}$ this is exactly the condition stated. If this is the case, then the number of letters of $\theta ^{(0,1)}$ is equal to the maximal choice of distinct maps $\theta _m \mathrm {pr_1}\times \theta _{m+1} \mathrm {pr_1}$, $1\le m <\ell -1$ (the size of $I_\theta$) times the size of the image of one of them, which is $|{{\mathcal {A}}}|$. $\square$

We go through some of the examples in [12]. The canonical outer encoding of any aperiodic 2-letter bijective substitution is an inner encoded substitution, because for those ${{\mathcal {A}}}^{(2)} = {{\mathcal {A}}}\times \text{\AA }= \{aa,ab,ba,bb\}$, $S_\theta = {\mathbb {Z}}/2{\mathbb {Z}}= \{{{\textbf{1}}},{\mathfrak {f}}\}$ and $I_\theta = \{{{\textbf{1}}},{\mathfrak {f}}\}$, where ${\mathfrak {f}}$ interchanges *a* with *b*. Indeed, this implies that the inner encoding is given by the partition $\{\{aa,bb\},\{ab,ba\}\}$. The simplest example is the Thue-Morse substitution

$$\begin{aligned} \theta : a&\mapsto abba \hspace{3em} b \mapsto baab \end{aligned}$$

and the inner encoded substitution associated to $\theta ^{(0,1)}$ is the well known period doubling substitution

$$\begin{aligned}  ^c\theta ^{(0,1)} : A&\mapsto ABAA \hspace{3em} B \mapsto ABAB \end{aligned}$$

which has a coincidence.

Our next example, from [12, Section 6.3], is

$$\begin{aligned} \theta : a&\mapsto abcca \hspace{3em} b \mapsto babab \hspace{3em} c \mapsto ccabc. \end{aligned}$$

It does not satisfy (7), as ${{\mathcal {A}}}^{(2)}$ has five letters and five is a prime number. Thus the substitution shift does not have an aperiodic almost automorphic factor.

As our last example, from [12, Section 6.3] has nontrivial height, we go through the required details carefully.

#### Example 8

The substitution

$$\begin{aligned} \theta : a&\mapsto abadcba \hspace{3em} b \mapsto badcbab \hspace{3em} c \mapsto cdcbadc \hspace{3em} d \mapsto dcbadcd \end{aligned}$$

has height 2. To find out whether the shift generated by this substitution has a proper almost automorphic factor, then, as it has height 2, we move to its pure base by Proposition 10. This is given by:

$$\begin{aligned}\tilde{\theta }:0&\mapsto 3 0 1 0 1 0 2 \hspace{3em} 1 \mapsto 2 1 0 1 0 1 3 \hspace{3em} 2 \mapsto 2 1 0 2 1 0 2 \hspace{3em} 3 \mapsto 3 0 1 3 0 1 3\end{aligned}$$

where each of the four symbols in the new substitution represents a two-letter-word from the original (respectively, 0, 1, 2, 3 correspond to [*ad*], [*cb*], [*cd*], [*ab*]). By inspection, we see that the minimal sets of this new substitution are disjoint, and thus its coincidence partition is given by ${\mathcal {P}}_{\tilde{\theta }}=\{\{0,1\},\{2,3\}\}$, which means that the map ${\tilde{F}}:X_{\tilde{\theta }}\rightarrow X_{\tilde{\eta }}$, whose local rule is a code and sends 0, 1 to *A* and 2, 3 to *B*, is a factor map to the aperiodic, primitive, almost automorphic substitution shift given by:

$$\begin{aligned} \tilde{\eta }:A&\mapsto B A A A A A B \hspace{3em} B \mapsto B A A B A A B. \end{aligned}$$

As in the construction description after Proposition 10, because the original substitution $\theta$ has height 2, to find a proper almost automorphic factor of $X_\theta$ we introduce a height-2 suspension $\eta$ of $\tilde{\eta }$ by “splitting” each symbol into two, moving from e.g. $A\mapsto BAAAAAB$ to $Aa\mapsto BbAaAaAaAaAaBb$, which is a concatenation of two length 7 words. The new substitution, almost automorphic by construction, is

$$\begin{aligned} \eta :A&\mapsto B b A a A a A \hspace{2.5em} B \mapsto B b A a A a B \hspace{2.5em} \\ a&\mapsto a A a A a B b \hspace{2.98em} b \mapsto b A a A a B b. \end{aligned}$$

The previously defined map ${\tilde{F}}$ induces a factor map $F:X_\theta \rightarrow X_\eta$. To define it explicitly, we use the fact that each element of $\{0,1,2,3\}$ corresponds to a two-letter word in $X_\theta$ and is mapped to either *A* or *B*, which also corresponds to the two-letter words *Aa* or *Bb* in $X_\eta$, so we expect *F* to map any instance of, say, *ad* in some $x\in X_\theta$ to the word *Aa* in the corresponding $F(x)\in X_\eta$. We can accomplish this by giving *F* left- and right-radius 1; accordingly, its local rule will be:

$$\begin{aligned} aba&\mapsto b&adc&\mapsto a&bab&\mapsto B&bad&\mapsto A \\ cba&\mapsto a&cdc&\mapsto b&dcb&\mapsto A&dcd&\mapsto B. \end{aligned}$$

### Veech towers

Let $F:(X,T)\rightarrow (Y,S)$ be a factor map, and let $E=\{ (x,x'): F(x)=F(x')\}$. We say that *F* is *isometric* if there is a continuous $R: E\rightarrow {\mathbb {R}}$ which is invariant under $T\times T$ and such that the restriction of *R* to any *F*-fibre $F^{-1}(y)\times F^{-1}(y)$ defines a metric on $F^{-1}(y)$. We say that (*X*, *T*) is an almost isometric extension ($\mathcal{A}\mathcal{I}$-extension for short) of (*Z*, *R*) if it has a factor $F:(X,T)\rightarrow (Y,S)$ and (*Y*, *S*) has a factor $\pi :(Y,S)\rightarrow (Z,R)$ such that *F* is isometric and $\pi$ is almost one-to-one. Veech’s theorem [19] tells us that minimal systems with a residual set of distal points have an almost automorphic extension which is an $\mathcal{A}\mathcal{I}$-*flow*, that is, which is the top system of a tower of $\mathcal{A}\mathcal{I}$ extensions of the trivial system $(pt,\textrm{id})$ containing only one point.

Veech’s theorem applies to primitive constant length substitution shifts $(X_\theta ,\sigma )$. A natural question is when $(X_\theta ,\sigma )$ is an $\mathcal{A}\mathcal{I}$-flow? In this case, can we exhibit a tower of $\mathcal{A}\mathcal{I}$-extensions for them? And if they are not an $\mathcal{A}\mathcal{I}$-flow can we find an almost one-to-one extension with its tower of $\mathcal{A}\mathcal{I}$-extensions? We do not answer these questions completely, but explain how our results on the existence of certain factors sheds light on them.

We recall Martin’s characterisation of when $(X_\theta , \sigma )$ is an AI-extension of its maximal equicontinuous factor. For ease of notation we state only the version for trivial height; for details of the general case, see [15, Theorem 8.08].

#### Theorem 11

Let $\theta$ be a bijective, primitive aperiodic substitution of length $\ell$ and of trivial height. The following are equivalent:

1. The cover ${\mathcal {U}}_{\theta ^{(0,1)}}$ is a partition.
2. $X_\theta$ is an almost isometric extension of its maximal equicontinuous factor.

Note that by Proposition 6, the first condition of the theorem is equivalent to the statement that the canonical outer encoding $^c\theta ^{(0,1)}$ is an aperiodic inner encoding.

Martin’s theorem is concerned with isometric extensions of almost automorphic substitutions, and one must distinguish this from characterising when a substitution has a proper almost automorphic factor. We give next an example of a bijective substitution $\theta$ which has an aperiodic almost automorphic factor $\eta$, but where the factor map $F:X_\theta \rightarrow X_\eta$ is not an isometry.

#### Example 9

Consider the following bijective substitution

$$\begin{aligned} \theta : a&\mapsto abbdb \hspace{3em} b \mapsto baaca \hspace{3em} c \mapsto cddbc \hspace{3em} d \mapsto dccad. \end{aligned}$$

The collaring $\theta ^{(0,1)}$ is a substitution on a 14-letter alphabet whose minimal sets are

$$\begin{aligned} A&:= \{(aa),(bb),(cc),(dd)\}&B&:=\{(ab),(ba),(cd),(dc)\}\\ C&:=\{(ac),(bd),(ca),(db)\}&D&:=\{(ad),(bc),(ca),(db)\}\\ E&:=\{(ab),(ba),(cc),(dd)\}&F&:=\{(aa),(bb),(cd),(dc)\}, \end{aligned}$$

so that ${\mathcal {U}}_{ \theta ^{(0,1)} }$ is not a partition. We have

$$\begin{aligned} {\mathcal {P}}_{ \theta ^{(0,1)} }&= \left\{ \{(aa),(ab), (ba), (bb), (cc), (cd), (dc), (dd) \} , \right. \\&\qquad \left. \{(ac),(ad), (bc), (bd), (ca), (db) \} \right\} \end{aligned}$$

consists of two sets, and the canonical inner encoding is

$$\begin{aligned} \eta : 0&\mapsto 00110 \hspace{3em} 1 \mapsto 00111 \end{aligned}$$

which is aperiodic. Furthermore the factor map from $X_\theta$ to $X_\eta$ is not isometric, sending eight $\theta$-fixed points to one fixed point for $\eta$, and six to the other. Finally, note that the canonical outer encoding

$$\begin{aligned}  ^c\theta ^{(0,1)}: A&\mapsto BACDE \hspace{3em} B \mapsto BACDF \hspace{3em} C \mapsto BACDD \\ D&\mapsto BACDC \hspace{3em} E \mapsto BACDA \hspace{3em} F \mapsto BACDB \end{aligned}$$

is not an inner encoding, but projects to the nontrivial inner encoding $\eta$ via the code $A,B,E,F\mapsto 0$, $C,D\mapsto 1$.

Martin’s results implies that bijective substitutions of trivial height which satisfy the condition of Theorem 11 are $\mathcal{A}\mathcal{I}$-flows having the following Veech tower of $\mathcal{A}\mathcal{I}$ extensions

$$\begin{aligned} X_\theta {\mathop {\rightarrow }\limits ^{F_{\beta _\theta }}}X_{ ^c\theta ^{(0,1)}} {\mathop {\rightarrow }\limits ^{F}}{\mathbb {Z}}_\ell {\mathop {\rightarrow }\limits ^{O}}\{ pt \} \end{aligned}$$

Here $F_{\beta _\theta }$ and *O* are isometric while *F* is almost one-to-one. We do not know of any result which is similar to Martin’s theorem but holds for non-bijective constant length substitutions. Finding conditions which guarantee that $F_{\beta _\theta }$ is isometric for non-bijective substitutions remains an open question. We can however say that, if the inner encoded substitution associated to $\theta ^{(0,1)}$ is periodic, then $X_\theta$ is not an $\mathcal{A}\mathcal{I}$-flow. This is a direct consequence of Martin’s Theorem 20 which implies that if $X_\theta$ is an ${\mathcal {A}}{\mathcal {I}}$-flow then it must be an $\mathcal{A}\mathcal{I}$ extension of its maximal equicontinuous factor.

Lemanczyk and Müllner [14] found an elegant construction of a substitution $\zeta$ whose dynamical system $(X_\zeta ,\sigma )$ is an almost one-to-one extension of $(X_\theta ,\sigma )$ and which always factors onto the system $(X_{ ^c\theta },\sigma )$ defined by the outer encoded substitution $^c\theta$. This system has the tower of extensions

$$\begin{aligned} X_\zeta {\mathop {\rightarrow }\limits ^{F_1}}X_{ ^c\theta } {\mathop {\rightarrow }\limits ^{F_2}}{{\mathcal {G}}}{\mathop {\rightarrow }\limits ^{O}}\{ pt \} \end{aligned}$$

and is therefore a Veech tower if $F_1$ is isometric. As this is an interesting ansatz we explain some details from [14] using our notation. Given a primitive length $\ell$ substitution $\theta$ of trivial height, let $U_\theta$ be its collection of minimal sets. Define

$$\begin{aligned} {\mathcal {C}}:= \{ (a,M): M\in U_\theta , a \in M\}, \end{aligned}$$

and recall the canonical outer encoding $^c\theta$ of $\theta$. Define $\zeta :{\mathcal {C}}\rightarrow {\mathcal {C}}^{\ell }$ by

$$\begin{aligned}\zeta _i (a,M) =( \theta _i(a),  ^c\theta _i(M)).\end{aligned}$$

In [14] the outer encoded substitution is directly defined (without reference to the semigroup of the substitution) and called the synchronising substitution. Furthermore, $\zeta$ is what is called there the joining of $\theta$ with $^c\theta$.

#### Proposition 12

([14]) $(X_\zeta , \sigma )$ is an almost one-to-one extension of $(X_\theta ,\sigma )$.

#### Proof

The code $(a,M)\mapsto a$ defines a factor $F:X_\zeta \rightarrow X_\theta$. We claim that if $\theta$ has column rank *c*, so does $\zeta$. For each *i* and each $M\in U_\theta$, $|\theta _i(M)|= c$. This implies that $|\{\zeta _i(a,M): a\in M\}|= c$, so $\zeta$ has column rank at least *c*. Also, one can assume, by moving to a power of $\theta$ is necessary, that there are *i* and $M^*\in U_\theta$ such that $\theta _i({\mathcal {A}})=M^{*}\in U_\theta$. This implies that $\{ \zeta _i(a,M): a\in M, M\in U_\theta \}= \{ (a,M^{*}): a\in M^{*}\}$, so $\zeta$ has column rank at most *c*.

Furthermore if $z\in {\mathbb {Z}}_\ell$ and $\pi ^{-1}(z)= \{x^{(1)}, \dots , x^{(c)} \}$ is a regular fibre in $X_\theta$, then for each $x^{(i)}$, $(\phi ^{-1}( x^{(i)}))_n= ( x^{(i)}_n, \{x^{(1)}_n, \dots , x^{(c)}_n \} )$. Thus $X_\zeta$ is an almost one-to-one extension of $X_\theta$. $\square$

In Section 6 of [14] one finds an example with a factor map $F_1:X_\zeta \rightarrow X_{ ^c\theta }$ which is not isometric. This raises the following question which we leave for future work: When is the factor map $F_1$ isometric?

##  Factoring onto a bijective substitution

In this section, we characterise, using the semigroup $S_\theta$, when a substitution shift is almost bijective. First we restrict to the case where the factor map preserves the fixed point fibre. We state the two main theorems and prove them in the next section.

### Theorem 13

Let $\theta$ be a constant length substitution with column rank *c*. The following are equivalent:

1. $S_\theta$ has a unique minimal left ideal.
2. There is a bijective substitution $\eta$ on a *c*-letter alphabet which is an inner encoding of $\theta$.

If these conditions are satisfied then $\eta$ is uniquely determined by $\theta$.

As $\eta$ is an inner encoding, it is always primitive. The following result shows that $\eta$ has to be aperiodic and that the associated factor map $F_\beta :X_\theta \rightarrow X_\eta$ has to be almost one-to-one, except if $\theta$ has a coincidence.

### Theorem 14

Let $\theta$ be a primitive, aperiodic length-$\ell$ substitution with column rank *c* and height *h*. Suppose that there is a bijective substitution $\eta$ on a *c*-letter alphabet which is inner encoded by $\theta$. Then $\eta$ is periodic if and only if $c=h$. Moreover, if $c>h$ then the factor map $X_\theta \rightarrow X_\eta$ induced by the inner encoding is almost one-to-one.

In general, the existence of a bijective substitution factor for a substitution is not linked to the existence of a bijective substitution factor for its pure base; see Example 15. The issue here is that we characterise the existence of a bijective factor in terms of the semigroup ${\mathcal {S}}_\theta$, and the relationship between this semigroup and that of the pure base of $\theta$ is not clear.

### More preliminaries from semigroup theory

In order to prove the above theorems we need to further analyse sub-semigroups of the semigroup ${{\mathcal {F}}}(X)$ of maps from $X\rightarrow X$. Recall that the partition defined by a map $f:X\rightarrow Y$ is ${{\mathcal {P}}}_f =\{f^{-1}(y) | y\in Y\}$.

#### Definition 10

We say that a map $g:X\rightarrow X$ *preserves a partition* ${{\mathcal {P}}}\subset {{\mathcal {P}}}(X)$ if $g^{-1}({{\mathcal {P}}})\subset {{\mathcal {P}}}$.

Stated differently, let ${{\mathcal {P}}}= \{A_i|i\in I\}$, then *g* preserves ${{\mathcal {P}}}$ if for all $i\in I$ there is a unique $j\in I$ such that $g^{-1}(A_i)=A_j$. Note that *g* does not necessarily preserve ${{\mathcal {P}}}_g$.

#### Lemma 11

Let $g:X\rightarrow X$ preserve a partition ${{\mathcal {P}}}$. Then $\left. g^{-1}\right| _{{{\mathcal {P}}}}$ is injective and hence bijective if ${{\mathcal {P}}}$ is finite.

#### Proof

Let $A,B\in {{\mathcal {P}}}$. Suppose $g^{-1}(A)=g^{-1}(B)$ which means $A\cap \textrm{im}g = B\cap \textrm{im}g$. Since *A* and *B* are either equal or have empty intersection, $A\cap \textrm{im}g = B\cap \textrm{im}g$ is the case if $A= B$ or $A\cap \textrm{im}g=B\cap \textrm{im}g=\emptyset$. But $A\cap \textrm{im}g =\emptyset$ means $g^{-1}(A)=\emptyset$, a possibility which is excluded, as a partition does not contain the empty set. $\square$

#### Lemma 12

Let $g:X\rightarrow X$ and $f:X\rightarrow Y$. If ${{\mathcal {P}}}_f={{\mathcal {P}}}_{fg}$ then *g* preserves ${{\mathcal {P}}}_f$.

#### Proof

By assumption

$$\begin{aligned} \{f^{-1}(y) | y\in Y\} = \{g^{-1}(f^{-1}(y)) | y\in Y\} \end{aligned}$$

which says exactly that $g^{-1}({{\mathcal {P}}}_f) = {{\mathcal {P}}}_f$. $\square$

A map $p:X\rightarrow X$ is an idempotent if and only if it preserves its partition and maps each member $A\in {{\mathcal {P}}}_p$ to a single point in *A*.

#### Lemma 13

Let $p:X\rightarrow X$ be an idempotent. If *p* preserves ${{\mathcal {P}}}$ and $|{{\mathcal {P}}}|=|{{\mathcal {P}}}_p|<+\infty$ then ${{\mathcal {P}}}_p={{\mathcal {P}}}$.

#### Proof

As *p* maps each member $A\in {{\mathcal {P}}}_p$ to a single point in *A*, ${{\mathcal {P}}}_p$ is the finest partition preserved by *p*. Hence if *p* preserves ${{\mathcal {P}}}$ then it preserves the partition generated by ${{\mathcal {P}}}$ and ${{\mathcal {P}}}_p$ which can’t be finer than ${{\mathcal {P}}}_p$. Therefore $|{{\mathcal {P}}}|=|{{\mathcal {P}}}_p|<+\infty$ implies ${{\mathcal {P}}}_p={{\mathcal {P}}}$. $\square$

#### Lemma 14

Let $p,q:X\rightarrow X$ two idempotents. If *p* preserves ${{\mathcal {P}}}_q$ then $qp = q$.

#### Proof

Suppose *p* preserves ${{\mathcal {P}}}_q$. Then it also preserves the partition generated by ${{\mathcal {P}}}_p$ and ${{\mathcal {P}}}_q$. As ${{\mathcal {P}}}_p$ is the finest partition preserved by *p*, ${{\mathcal {P}}}_q$ must be coarser than ${{\mathcal {P}}}_p$ or ${{\mathcal {P}}}_q={{\mathcal {P}}}_p$ . It follows that $\left. p^{-1}\right| _{{{\mathcal {P}}}_q}$ is the identity map from ${{\mathcal {P}}}_q$ to itself. This implies $q^{-1}=p^{-1}q^{-1}$ hence $qp=q$. $\square$

The condition of the following lemma is satisfied for all compact right topological semigroups and so in particular for any finite semigroup.

#### Lemma 15

Let *S* be a sub-semigroup of ${{\mathcal {F}}}(X)$ which admits a kernel which contains an idempotent. The following are equivalent.

1. All elements of *S* preserve the partition defined by the idempotent.
2. *S* has a unique minimal left ideal, i.e., the kernel is left simple.

#### Proof

$1\Rightarrow 2$. Let $q\in S$ be a minimal idempotent such that all elements of *S* preserve ${{\mathcal {P}}}_q$. Let $p\in S$ be another minimal idempotent. By assumption *p* preserves ${{\mathcal {P}}}_q$. By Lemma 14 we have $qp=q$. Hence *q* lies in the minimal left ideal generated by *p*. Since all idempotents of a minimal left ideal generate the same left ideal, therefore *S* has a unique minimal left ideal.

$2\Rightarrow 1$. Let *p* be an idempotent in the unique minimal left ideal *L*. Let $s\in S$. Then *ps* lies in the kernel of *S* which coincides with *L*. By Lemma 22, ${{\mathcal {P}}}_{ps}={{\mathcal {P}}}_p$. By Lemma 12, *s* preserves ${{\mathcal {P}}}_p$. $\square$

#### Proof of Theorem 13

Suppose first that ${{\mathcal {S}}}_\theta$ contains a unique minimal left ideal. By Lemma 15 all its elements preserve a partition ${{\mathcal {B}}}= \{ A_1,\cdots , A_k\}$ defined by any element of that ideal. Note that *k* must be the column rank *c*. Define the length-$\ell$ substitution $\eta$ on the alphabet ${{\mathcal {B}}}$ as that having column maps $\eta _m$ such that

$$\begin{aligned} \eta ^{-1}_m:= \left. \theta ^{-1}_m\right| _{{{\mathcal {B}}}} \,. \end{aligned}$$

In other words, $\eta _m(A_i)$ is the unique $A_j$ which contains $\theta _m(a_i)$ for some $a_i\in A_i$. This is well-defined as $\theta _m$ preserves the partition ${{\mathcal {B}}}$. By Lemma 11$\eta$ is bijective. The code $\beta$ is given by $a\mapsto A_i$ for all $a\in A_i$.

To prove the converse of the statement, suppose now there is a bijective substitution with column maps $\eta _m$ on a *c*-letter alphabet ${{\mathcal {B}}}$ which is inner encoded by $\theta$, that is, there is a map $\beta :{{\mathcal {A}}}\rightarrow {{\mathcal {B}}}$ such that

$$\begin{aligned} \eta _m\circ \beta = \beta \circ \theta _m \end{aligned}$$

for all $m=0,\cdots ,\ell -1$. Since $\eta$ is bijective this implies that the partition ${{\mathcal {P}}}_\beta$ defined by $\beta$ must be the same as that defined by each $\beta \circ \theta _m$ and then also the same as that defined by any $\beta \circ f$ for any $f\in S_\theta$. By Lemma 12 all elements of $S_\theta$ must preserve ${{\mathcal {P}}}_\beta$. Let *p* be a minimal idempotent of $S_\theta$. By definition, its column rank is *c*, which is also the rank of $\beta$. Lemma 13 implies therefore that ${{\mathcal {P}}}_p={{\mathcal {P}}}_\beta$. Now, we conclude with Lemma 15 that ${{\mathcal {S}}}_\theta$ has a unique minimal left ideal.

The uniqueness of the $\eta _m$ follows from the fact that it is entirely determined by the $\theta _m$ and the partition induced from any idempotent of $S_\theta$. $\square$

#### Proof of Theorem 14

Suppose that $\eta$ is a bijective substitution on a *c*-letter alphabet which is inner encoded by $\theta$. Clearly, if $c=1$ then $\eta$ is periodic. Now suppose $c>1$.

It is one of the properties of the height *h* that there is a code $\beta : {{\mathcal {A}}}_\theta \rightarrow {\mathbb {Z}}/h{\mathbb {Z}}$ which induces a factor map $F_\beta :(X_\theta ,\sigma )\rightarrow ({\mathbb {Z}}/h{\mathbb {Z}},+1)$ such that $\beta (\theta (a))=\beta (\theta (b))$ if $\beta (a)=\beta (b)$ [5]. In other words the partition induced by $\beta$ satisfies the condition (2) of Lemma 3 and so defines a substitution $\eta '$ which is inner encoded by $\theta$. This substitution is periodic, as its substitution space is finite. If $c=h$ it satisfies the properties of Theorem 13 and hence must be (up to a renaming of its alphabet) equal to $\eta$.

Suppose now that $\eta$ is $h'$-periodic. Hence $(X_\eta ,\sigma )\cong ({\mathbb {Z}}/h'{\mathbb {Z}},+1)$ and ${\eta ^N}_{nh'} = \eta _0$ for all $n\in {\mathbb {N}}$ and *N* such that $\ell ^N>nh'$. Taking a power we may assume that $\eta _0={{\textbf{1}}}$. Clearly $1/h'$ is an eigenvalue of $(X_\eta ,\sigma )$, hence also of $(X_\theta ,\sigma )$. If $1/h'\in {\mathbb {Z}}_\ell$ then there are *n*, *N* such that $nh' = \ell ^N$. Hence, for all $0\le k<\ell$

$$\begin{aligned} {{\textbf{1}}}={\eta ^{N+1}}_{k\ell ^N} = {\eta ^N}_0\eta _k = \eta _k, \end{aligned}$$

which contradicts the primitivity of $\eta$, as $c > 1$. Hence $h'$ must divide the height *h* of $\theta$. As $\eta$ is bijective we have $c=|{{\mathcal {A}}}_\eta |\le |X_\eta |=h'\le h$.

We show that the factor map $F:X_\theta \rightarrow X_\eta$ induced by the inner encoding is almost one-to-one provided $\eta$ is aperiodic. Recall $\pi _\theta :X_\theta \rightarrow {\mathbb {Z}}_\ell$ and $\pi _\eta :X_\eta \rightarrow {\mathbb {Z}}_\ell$ are the factor maps onto the equicontinuous factor ${\mathbb {Z}}_\ell$ which map the fixed points of $\theta$ and $\eta$, resp., to 0. Then $\pi _\theta = \pi _\eta \circ F$. The regular points for $\theta$, are those $z\in {\mathbb {Z}}_\ell$ which have a fibre of size *c*, similarly for $\eta$. For both substitutions they are known to form a residual set. Hence their intersection is not empty. Let *z* be regular for both $\theta$ and $\eta$. Then *F* must restrict to a bijection on the corresponding fibre. Hence *F* is almost one-to-one. $\square$

#### Remark

It follows from Lemma 7 that our characterisation of when a substitution allows for an inner encoded bijective substitution is stable under taking powers.

#### Example 10

Consider the primitive aperiodic substitution

$$\begin{aligned} \theta :0&\mapsto 021 \hspace{3em} 2 \mapsto 201 \hspace{3em} 1 \mapsto 130 \hspace{3em} 3 \mapsto 310 \end{aligned}$$

which is easily checked to have column rank 2. The partition defined by the map $\theta _2$ is

$$\begin{aligned} {{\mathcal {P}}}_{\theta _2} = \{ \{0,2\}, \{1,3\}\}. \end{aligned}$$

It is easily seen to be preserved by all products of $\theta _i$’s. Hence $S_\theta$ has a unique minimal left ideal. The code

$$\begin{aligned} \beta :0,2&\mapsto a \hspace{3em} 1,3 \mapsto b \end{aligned}$$

gives rise to the inner encoded bijective aperiodic substitution

$$\begin{aligned} \eta :a&\mapsto aab \hspace{3em} b \mapsto bba. \end{aligned}$$

As an aside we mention that the substitution shift defined by $\theta$ has uncountably many irregular fibers and hence the factor map $F_\beta :X_\theta \rightarrow X_\eta$ is not one-to-one on uncountably many points.

### Factors which preserve the fixed point fibre

In Theorem 13, we characterised the substitution shifts which admit a bijective inner encoding. In this section, we extend this to characterising substitution shifts which admit a bijective substitution shift as a factor via a factor map which preserves the fixed point fibre.

#### Lemma 16

Let $n=(-l,r)$. If $S_{\theta ^{(n)}}$ has a unique minimal left ideal then $S_\theta$ has a unique minimal left ideal.

#### Proof

As $\theta$ is an inner encoding of the collared substitution $\theta ^{(n)}$, the semigroup $S_\theta$ is a homomorphic image of $S_{\theta ^{(n)}}$. The result follows therefore from Lemma 24. $\square$

#### Theorem 15

Let $\theta$ be a primitive aperiodic constant length substitution with height *h* and column rank $c>h$. The following are equivalent.

1. $(X_\theta , \sigma )$ is an almost one-to-one extension of a bijective substitution shift $(X_\eta ,\sigma )$ via a factor map *F* which preserves the fixed point fibre.
2. $(X_\theta , \sigma )$ factors onto a *c*-letter bijective substitution shift $(X_\eta ,\sigma )$ via a factor map *F* which preserves the fixed point fibre.
3. $S_\theta$ has a unique minimal left ideal.

#### Proof

To show that (1) implies (2), we only have to show that the bijective substitution $\eta$ is on *c* letters. As *F* is almost one-to-one, $X_\theta$ and $X_\eta$ must have the same maximal equicontinuous factor, hence the same height and therefore also the same column rank. For a bijective substitution the column rank equals the size of its alphabet.

To see that (2) implies (3), suppose that $F:X_\theta \rightarrow X_\eta$ is a factor map which preserves the fixed point fibre and where $\eta$ is a bijective substitution on *c* letters. By Corollary 2(2) there exist $n=(-l,r)$, $l,r\le 1$, $p\ge 1$, an inner encoding $(\eta ',\beta )$ of ${\theta ^{(n)}}^p$, and a code $\tau ':{{\mathcal {A}}}_{\eta '} \rightarrow {{\mathcal {A}}}_{\eta }$ such that $F_{\tau '}:X_{\eta '}\rightarrow X_\eta$ is a conjugacy. As each of *F* and $F_\beta$ preserve the fixed point fibres, $F_{\tau '}$ must also preserve the fixed point fibre. By Lemma 2$(\eta ^p,\tau ')$ is an inner encoding of $\eta '$. Hence $(\eta ^p,\tau '\circ \beta )$ is an inner encoding of ${\theta ^{(n)}}^p$. By assumption $\eta$ and also $\eta ^p$ is a substitution on *c* letters. Now Theorem 13 implies that $S_{{\theta ^{(n)}}^p}$ has a unique minimal left ideal. By Lemmata 7 and 16, $S_\theta$ has a unique minimal left ideal.

Finally we come to (3) implies (1). Suppose that $S_\theta$ has a unique minimal left ideal. By Theorem 13 there exists a bijective substitution on a *c* letter alphabet which is an inner encoding of $\theta$. By Lemma 2 the associated factor map preserves the fixed point fibre. By Theorem 14 the factor map is almost one-to-one. $\square$

We remark that Theorems 13 and 15 imply that all factor maps to a bijective substitution which fix the fixed point fibre must have radius zero.

#### Example 11

The substitution shift $X_\theta$ of Example 1 does not admit a factor map onto an aperiodic bijective substitution shift which fixes the fixed point fibre. Indeed, any aperiodic bijective substitution shift which is a factor of $X_\theta$ must have 2 letters, as the column rank of $\theta$ is 2, and this is excluded as the semigroup $S_\theta$ has two minimal left ideals.

### Bijective factors which do not preserve the fixed point fibre

In this section we extend Theorem 15 to substitutions which have a bijective substitution factor via a factor map which does not send fixed points to fixed points. To do this we need to recall a little more information on the arithmetic properties of factor maps between substitutions.

### Factors of substitution shifts and their $\kappa$-values

Let $(X,\sigma )$ and $(Y,\sigma )$ be infinite minimal shifts with a common equicontinuous factor $({\mathcal {G}},R)$ and fixed equicontinuous factor maps $\pi _X:X\rightarrow {\mathcal {G}}$ and $\pi _Y:Y\rightarrow {\mathcal {G}}$. Let $\textrm{Fac}(X,Y)$ be the collection of factor maps from $(X,\sigma )$ to $(Y,\sigma )$. Following [4] we define the map $\kappa : \text{ Fac }(X,Y) \rightarrow {\mathcal {G}}$ through

$$\begin{aligned} \kappa (F):= \pi _Y(F( x))- \pi _X(x). \end{aligned}$$

$\kappa (F)$ is also called the $\kappa$-value of *F*. By minimality, it does not depend on *x*. But it depends on the choice of $\pi _X$ and $\pi _Y$ and in the framework of substitution shifts, which we consider here, we continue to choose them in such a way that the fixed points of the substitutions are mapped to 0. By [4, Theorem 3.3] the map $\kappa$ satisfies

1. if $(Z,\sigma )$ is another shift with equicontinuous factor ${{\mathcal {G}}}$ and $F \in \text{ Fac } (X,Y)$, $G \in \text{ Fac }(Y,Z)$ then
  $$\begin{aligned} \kappa (G\circ F)=\kappa (G)+\kappa (F), \end{aligned}$$
2. if $\min _{g\in {\mathcal {G}}}|\pi _X^{-1}(g)|=\min _{g\in {\mathcal {G}}}|\pi _Y^{-1}(g)|=c<\infty$ then $\kappa :\text{ Fac }(X,Y)\rightarrow {{\mathcal {G}}}$ is at most *c*-to-one and
  $$\begin{aligned} \{ g \in {{\mathcal {G}}}: |\pi _Y^{-1}(g)|>c \}\subset \{g \in {{\mathcal {G}}}: |\pi _X^{-1}(g)|>c \} + \kappa (F) \end{aligned}$$
  for all $$F\in \text{ Fac }(X,Y)$$.

If $F:X_\theta \rightarrow X_\eta$ is a factor map between two length-$\ell$ substitution shifts, then $\kappa (F)=0$ if and only if $\theta$-fixed points are mapped to $\eta$-fixed points, i.e., if *F* preserves the fixed point fibre. In what follows, we will take ${\mathcal {G}}={\mathbb {Z}}_\ell$, i.e.,

$$\begin{aligned}\kappa : \text{ Fac }(X_\theta , X_\eta ) \rightarrow {\mathbb {Z}}_\ell ,\end{aligned}$$

even if $\theta$ has nontrivial height, so that the *c* in (2) above is the column rank of $\theta$ and $\eta$.

The following adaptation of [4, Proposition 3.24] tells us that $\kappa$-values of factors between two constant length substitution shifts can only take certain values. We say that $z\in {\mathbb {Z}}_\ell$ is *rational* if it is eventually periodic. This naming is motivated by the fact that if $z\in {\mathbb {Z}}_\ell$ is eventually periodic, then it is the $\ell$-adic expansion of a rational number.

#### Proposition 16

Let $\theta$ and $\theta '$ be primitive, aperiodic length-$\ell$ substitutions and $F\in \text{ Fac }(X_\theta ,X_{\theta '})$. Then $\kappa (F)$ is rational.

#### Proof

In the case where the factor map is a conjugacy, and the heights of $\theta$ and $\eta$ are trivial, the statement is [4, Proposition 3.24]. That proposition can be readily modified to hold for the case in which the heights of $\theta$ and $\eta$ are not necessarily trivial, namely by taking for ${{\mathcal {G}}}$ not the maximal equicontinuous factor but ${\mathbb {Z}}_\ell$.

If the factor map *F* is not a conjugacy we apply Proposition 5 to $Y=X_{\theta '}$ from which we deduce that $\kappa (F) = \kappa (F_{\tau })$ as, by Lemma 2, $F_\imath$ preserves the fixed point fibre, c.f. Figure 2 with $Y=X_{\theta '}$. Going over to $\theta ^{(n)}$ if necessary we may thus assume that $F=F_\tau$ for some code $\tau :{\mathcal {A}}_\theta \rightarrow {\mathcal {A}}_{\theta '}$.

By Theorem 4 applied to $Y=X_{\theta '}$ there is an inner encoding $(\eta _\tau , \beta _\tau )$ of $\theta$, and a code $\tau ':{\mathcal {A}}_{\eta _\tau }\rightarrow {\mathcal {A}}_{\theta '}$ such that $F_{\tau '}:X_\eta \rightarrow X_{\theta '}$ is a conjugacy, c.f. Figure 1 with $Y=X_{\theta '}$. As, by Lemma 2, $F_{\beta _\tau }$ preserves the fixed point fibre, we have $\kappa (F_\tau )=\kappa (F_{\tau '})$. Now we apply [4, Proposition 3.24] to the conjugacy $F_{\tau '}$ to obtain that $\kappa (F_{\tau '})$ and hence $\kappa (F_\tau )$ is rational. $\square$

If $F:X_\theta \rightarrow X_\eta$ is a factor map onto a bijective substitution shift with $\kappa (F)=m\in {\mathbb {Z}}$, then ${{\tilde{F}}}:=\sigma ^{-m}\circ F: X_\theta \rightarrow X_\eta$ is also a factor map onto a bijective substitution and, as $\kappa ({{\tilde{F}}})=0$, we can apply Theorem 15 to conclude that $S_\theta$ has a unique left minimal ideal. In other words, Theorem 15 extends verbatim to the case in which the $\kappa$-value of the factor map is an integer.

However, the following example shows that there exist factor maps $F:X_\theta \rightarrow X_\eta$ where $\eta$ is bijective, where $\kappa (F)\not \in {\mathbb {Z}}$, and where ${\mathcal {S}}_\theta$ has more than one minimal left ideal.

#### Example 12

Take the following two substitutions:

$$\begin{aligned} \eta :a&\mapsto abcba&\theta :0&\mapsto 35203 \hspace{3em} 3 \mapsto 41534 \\ b&\mapsto bcacb&1&\mapsto 35214 \hspace{3em} 4 \mapsto 02140\\ c&\mapsto cabac&2&\mapsto 41520 \hspace{3em} 5 \mapsto 02153. \end{aligned}$$

Clearly $\eta$ is bijective. The words of length two in ${\mathcal {L}}_\eta$ are

$$\begin{aligned} \{ab,ac,ba,bc,ca,cb\},\end{aligned}$$

and by mapping (*ab*), (*ac*), (*ba*), (*bc*), (*ca*), (*cb*) to 0, 1, 2, 3, 4, 5, respectively, it can be verified that $\theta =\eta ^{(+1)}$. Thus $X_\theta$ factors onto the bijective substitution shift $X_\eta$. However, ${{\mathcal {S}}}_{\theta }$ does *not* have a unique minimal left ideal. Indeed, it has two minimal left ideals, one associated to the partition $\{ \{ 0,1\}, \{2,3 \},\{4,5 \}\}$, and the other to the partition $\{ \{ 0,5\}, \{1,3 \},\{2,4 \}\}$; see Lemma 22 which shows how the ${{\mathcal {L}}}$-class constituting a minimal left ideal is associated to a partition. This does not contradict Theorem 15 because, as we will see below, the conjugacy $F_\tau :X_{\theta }\rightarrow X_\eta$ which is given by the code

$$\begin{aligned} \tau :0,1&\mapsto a&2,3&\mapsto b&4,5&\mapsto c \end{aligned}$$

is a factor map with nonzero $\kappa$-value. In particular, Lemma 17 will tell us that $\kappa ( F_\tau )= -1/4= -1/(\ell -1)$.

We now show how to “correct" such factor maps to obtain factor maps with $\kappa$-value 0. Recall the *k*-shifted extension of $\theta$ from Definition 2.

#### Lemma 17

Let $\theta : {\mathcal {A}} \rightarrow {\mathcal {A}}^{\ell }$ be an aperiodic primitive length-$\ell$ substitution, and let $\zeta :=\theta ^{(+ k)}$ be its *k*-shifted extension, with $0 \leqslant k < \ell$. Then there is a conjugacy $F: X_{\zeta } \rightarrow X_{\theta }$ with $\kappa (F) = \frac{k}{ 1-\ell }$.

#### Proof

Recall from Corollary 1 that $F_\imath : X_\zeta \rightarrow X_\theta$ is a conjugacy. We consider its inverse $F_\imath ^{-1}$. By iteration of the equation of Corollary 1 we obtain

$$\begin{aligned} \zeta ^n \circ F^{-1} = F^{-1} \circ (\sigma ^k\circ \theta )^n= F^{-1}\circ \sigma ^{k\frac{1-\ell ^n}{1-\ell }} \circ \theta ^n. \end{aligned}$$

Let *u* be a fixed point of $\theta$. Then $v = \lim _{n\rightarrow \infty } \zeta ^n(F_\imath ^{-1}u)$ is a fixed point of $\zeta$ (we assume that the periodic points of $\zeta$ are fixed points). Then, by continuity of $F_\imath ^{-1}$,

$$\begin{aligned} v = F_\imath ^{-1} \circ \lim _{n\rightarrow \infty } \sigma ^{k\frac{1-\ell ^n}{1-\ell }} (u), \end{aligned}$$

so that $\kappa ( F_\imath ^{-1} ) = \frac{-k}{1-\ell }$. The result follows. $\square$

#### Corollary 6

Let $\theta : {\mathcal {A}} \rightarrow {\mathcal {A}}^{\ell }$ be an aperiodic primitive length-$\ell$ substitution, and let $\frac{p}{q}\in {\mathbb {Z}}_\ell$ with *p*, *q* coprime. Then there exists some substitution $\zeta$ and a conjugacy $F: X_{\zeta } \rightarrow X_{\theta }$ that satisfies $\kappa (F)= \frac{p}{q}$.

#### Proof

As shown in Lemma 17, if $0\le k<\ell$ and $\zeta = \theta ^{(+k)}$, then $\kappa (F_\imath ) = \frac{k}{1 - \ell }$. Similarly, if we replace $\theta$ by $\theta ^m$ for some value of *m* and take $\zeta = (\theta ^m)^{(+k)}$, the corresponding factor map has $\kappa$-value $\frac{k}{1 - \ell ^m}$, where we can take *k* to be any value between 0 and $\ell ^m - 1$.

To prove the general statement, suppose first that $- q < p \leqslant 0$, and that *p* and *q* have no common divisors. As we assume that $p/q\in {\mathbb {Z}}_\ell$, then *q* and $\ell$ are coprime, so that $\ell ^{\varphi (q)} \equiv 1 \pmod {q}$, where $\varphi$ is Euler’s totient function. Thus $\ell ^{\varphi (q)} - 1 = hq$ for some $h > 0$. Since $- q < p \leqslant 0$, we have $0 \leqslant - hp< hq < \ell ^{\varphi (q)}$, so we can take $\zeta = (\theta ^{\varphi (q)})^{( + (-hp))}$, and the conjugacy $F_\imath : X_{\zeta } \rightarrow X_{\theta }$ will satisfy:

$$\begin{aligned} \kappa (F_\imath ) = \frac{-hp}{1 - \ell ^{\varphi (q)}} = \frac{-hp}{-hq} = \frac{p}{q}, \end{aligned}$$

as desired.

For the general case, it is enough to note that $\frac{p}{q} = M + \frac{p_0}{q}$ for some integer *M* and $- q < p_0 \leqslant 0$, where $p_0$ will also be coprime to *q*, so it suffices to take $\zeta = (\theta ^{\varphi (q)})^{(+(- hp_0))}$, as above, and $\sigma ^M \circ F_\imath$ as the desired conjugacy. $\square$

Define, for a substitution with column rank *c*,

$$\begin{aligned} {\mathcal {F}}_\theta := \{ w=w_k \ldots w_1 \in ({\mathbb {Z}}/ \ell {\mathbb {Z}})^+: |\theta _{w_1}\circ \ldots \circ \theta _{w_k}({\mathcal {A}})|=c \}. \end{aligned}$$

Note that if ${\mathcal {F}}_\theta$ contains a length *k* word, then $\theta ^k$ has a column with *c* elements.

#### Lemma 18

Let $\theta$ be a constant length substitution. Let $(\eta ,\beta )$ be an inner encoding of $\theta$. The length $j(\eta )$ of the shortest word in ${{\mathcal {F}}}_\eta$ is bounded by the length $j(\theta )$ of the shortest word in ${{\mathcal {F}}}_\theta$.

#### Proof

Let $U_\theta = \{\textrm{im}f:f\in \ker S_\theta \}$. We first show that $U_\eta = \beta (U_\theta )$. Indeed, by Lemma 24 we have $\ker S_\eta = \beta (\ker S_\theta )\beta ^{-1}$ and hence

$$\begin{aligned} U_\eta = \{\textrm{im}f:f\in \ker S_\eta \} = \{\textrm{im}\beta \circ g: g \in \ker S_\theta \}. \end{aligned}$$

By definition, $w_k \ldots w_1$ is a word in ${{\mathcal {F}}}_\theta$ if and only if $\theta _{w_1}\circ \ldots \circ \theta _{w_k}({\mathcal {A}})\in U_\theta$. Now, by the above,

$$\begin{aligned} \eta _{w_1}\circ \ldots \circ \eta _{w_k}({\mathcal {B}})= \beta \circ \theta _{w_1}\circ \ldots \circ \theta _{w_k}({\mathcal {A}})\in \beta (U_\theta ) = U_\eta . \end{aligned}$$

This implies ${{\mathcal {F}}}_\theta \subset {{\mathcal {F}}}_\eta$ and hence $j(\eta )\le j(\theta )$. $\square$

#### Lemma 19

Let $\theta : {\mathcal {A}} \rightarrow {\mathcal {A}}^{\ell }$ be an aperiodic primitive length-$\ell$ substitution, and let $F:X_\theta \rightarrow X_\zeta$ be a factor map. If ${\mathcal {F}}_\theta$ contains a word of length *j*, then $n\kappa (F) \in {\mathbb {Z}}$ for some $0\le n\le (\ell -1) ( \ell ^{j}-1)$.

#### Proof

In the case where the factor map is a conjugacy, the statement is [4, Proposition 3.24], with the appropriate modifications to account for height, as discussed in the proof of Proposition 16. Otherwise we proceed as in the proof of Proposition 16 and apply Proposition 5 to $Y=X_{\theta '}$. We may thus assume that $F=F_\tau$ for some code $\tau :{\mathcal {A}}_\theta \rightarrow {\mathcal {A}}_{\zeta }$.

Theorem 4 tells us that there is a pair $(\eta , \beta )$ which is inner encoded by $\theta$, and a code $\tau ':{\mathcal {A}}_{\eta }\rightarrow {\mathcal {A}}_{\zeta }$ such that $F_{\tau '}:X_\eta \rightarrow X_{\zeta }$ is a conjugacy, and $\kappa (F_\tau )= \kappa (F_{\tau '})$. Then again by [4, Proposition 3.24], the denominator of $\kappa (F_{\tau '})$ (always assuming that it is coprime to the numerator) is at most $(\ell -1) ( \ell ^{j}-1)$, where $j=j(\eta )$ is the length of the shortest word in ${\mathcal {F}}_\eta$. Now by Lemma 18, we have that $j(\eta )\le j(\theta )$. Thus the denominator of $\kappa (F_{\tau '})$ is at most $(\ell -1) ( \ell ^{j(\theta )}-1)$. Since $\kappa (F_{\tau '})= \kappa (F_\tau )$, the result follows. $\square$

#### Theorem 17

Let $\theta$ be an aperiodic primitive constant length-$\ell$ substitution with column rank $c > 1$. The following are equivalent:

1. $X_\theta$ is an almost one-to-one extension of an aperiodic bijective length-$\ell$ substitution shift.
2. There exists $0\le n,k$ such that the semigroup $S_{(\theta ^n)^{(+k)}}$ contains a unique minimal left ideal. Moreover, if *j* is the length of the shortest word in ${\mathcal {F}}_\theta$, then $n\le (\ell -1)(\ell ^j -1)$ and $0\le k \le \ell ^n -1$.

#### Proof

Let $F:(X_\theta ,\sigma )\rightarrow (X_\eta ,\sigma )$ where $\eta$ is bijective, defined on a *c*-letter alphabet. By Proposition 16, $\kappa (F)$ is rational, and by Lemma 19, $\kappa (F)=p/q$ where $1\le q\le (\ell -1)(\ell ^j -1)$, where *j* is the length of the shortest word in ${\mathcal {F}}_\eta$. Using $\varphi (q) \le (\ell -1)(\ell ^j -1)$, and taking a shift if necessary, Corollary 6 tells us that there is $n\le (\ell -1)(\ell ^j -1)$ and $0\le k \le \ell ^{n}$, and a conjugacy ${{\tilde{F}}}:(X_{(\theta ^n)^{(+k)}},\sigma )\rightarrow (X_\theta ,\sigma )$ such that $\kappa (F)+\kappa ({{\tilde{F}}})\in {\mathbb {Z}}$. As $\kappa ( F\circ {{\tilde{F}}})\in {\mathbb {Z}}$, then $S_{(\theta ^n)^{(+k)}}$ must have a unique minimal left ideal by Theorem 15.

For the converse, if $S_{(\theta ^n)^{(+k)}}$ has a unique minimal left ideal, then by Theorem 15, $(X_{(\theta ^n)^{(+k)}},\sigma )$ factors dynamically onto a bijective substitution shift. As $(X_{(\theta ^n)^{(+k)}},\sigma )$ is conjugate to $(X_\theta , \sigma )$, the result follows. $\square$

The following corollary follows from a straightforward application of the comments in Sect. 3.5 and underlines an interest in finding bijective factors.

#### Corollary 7

Suppose that $X_\theta$ is almost bijective with corresponding bijective substitution $\eta$ such that ${{\mathcal {U}}}_{\eta ^{(0,1)}}$ is a partition of ${{\mathcal {A}}}_\eta ^{(2)}$. Then $X_{\eta }$ factors isometrically onto a proper almost automorphic substitution shift $X_\zeta$ and

$$\begin{aligned} X_{\theta } {\mathop {\rightarrow }\limits ^{F_1}}X_{\eta } {\mathop {\rightarrow }\limits ^{F_2}}X_\zeta {\mathop {\rightarrow }\limits ^{F_3}}{\mathbb {Z}}_\ell {\mathop {\rightarrow }\limits ^{O}}\{pt\} \end{aligned}$$

is a Veech tower for $X_\theta$, $F_1,F_3$ being almost one-to-one and $F_2,O$ isometric.

#### Example 13

We return to the Rudin Shapiro substitution $\theta$, in Example 5. One verifies that the length *j* of the shortest forbidden word in ${\mathcal {F}}_\theta$ is $j=1$, so by Theorem 17, $X_\theta$ factors onto a bijective substitution shift if and only if $S_{\theta ^{(+k)}}$ contains a unique minimal left ideal, where $0\le k \le 1$. One can verify that each of $S_{\theta ^{(+0)}}$ and $S_{\theta ^{(+1)}}$ have two minimal left ideals. Therefore $\theta$ does not factor onto a bijective substitution shift.

We remark that if $(\ell -1)(\ell ^j -1)>1$, then by inspecting the proof of the second part of Theorem 17, namely using $\varphi (q)\le q-1$ if $q>1$, we can improve the bound to $n\le (\ell -1)(\ell ^j -1) -1$ and $0\le k \le \ell ^n -1$. We will use this in the examples below.

#### Example 14

We return to Example 12, with $\theta$ and $\eta$ defined there. We take $\zeta := \theta ^{(+3)}$, and we claim that $\zeta$ has a unique minimal ideal. The reason why we make this choice $\theta ^{(+3)}$ is that, by Lemma 17, the natural conjugacy $G: X_\zeta \rightarrow X_\theta$ satisfies $\kappa (G) = -3/4$. Then $\kappa (\sigma \circ F)= 3/4$, and $F\circ \sigma \circ G: X_\zeta \rightarrow X_\eta$ has $\kappa$-value zero. Note though that $F \circ \sigma \circ G$ has right radius one, so we will need to work with $X_{\zeta ^{(2)}}$. Theorem 15 guarantees that ${\mathcal {S}}_{\zeta ^{(2)}}$ has a unique minimal left ideal, and Lemma 16 guarantees that so also does ${\mathcal {S}}_{\zeta }$. Indeed, to define $\zeta$, we simultaneously list and code the words of length two in ${\mathcal {L}}_\theta$:

$$\begin{aligned} A&=02, \hspace{5em} B=03, \hspace{5em} C=14, \hspace{5em} D=15,\hspace{5em}\\ E&=20, \hspace{5em}F=21, \hspace{5em} G=34, \hspace{5em} H=35, \hspace{5em}\\ I&=40, \hspace{5em} J=41,\hspace{5em} K=52, \hspace{5em}L=53; \end{aligned}$$

it then can be verified that the 3-shifted extension $\zeta :=\theta ^{(+3)}$ of $\theta$ has a unique minimal left ideal, generated by the partition $\{\{ A,B, K,L\}, \{ C,D,G,H\}, \{E,F,I,J \}\}$.

We end with an example that shows that if a substitution shift has a bijective substitution factor, this does not imply that its pure base has a bijective substitution factor, illustrating that we cannot reduce to work with the pure base.

#### Example 15

Consider the following substitution on $\{a,{\bar{a}},b,c,d,e,f\}$:

$$\begin{aligned}\theta :a&\mapsto a d c \hspace{5em} {\bar{a}} \mapsto {\bar{a}} d c \hspace{5em} b \mapsto b e a \hspace{5em} c \mapsto c f b \\ d&\mapsto d {\bar{a}} e \hspace{5em} e \mapsto e b f \hspace{5em} f \mapsto f c d. \end{aligned}$$

It is routine to check that this substitution is primitive and has height 2. We can define a factor map $F_\tau :X_\theta \rightarrow X_\eta$, which has local rule $\tau : \{a,{\bar{a}},b,c,d,e,f\}\rightarrow \{a,b,c,d,e,f\}$, with $\tau (a)= \tau ({\bar{a}})$, and $\tau$ is the identity otherwise; $\eta$ is the resulting inner encoding. It can be verified that $\eta$ is bijective. As such, we can check that $S_\theta$ has indeed a unique minimal left ideal, satisfying the conditions of Theorem 13.

Given that both $\theta$ and $\eta$ have height 2, one may proceed as in the proof of Proposition 10 and define a factor map between the pure bases $\tilde{\theta }$ and $\tilde{\eta }$ of both substitutions, but, in general, the property of being bijective is *not* a conjugacy invariant. In fact, $\tilde{\eta }$ is not a bijective substitution. Furthermore, it cannot be taken to be conjugate to one: it can be verified that the maximal equicontinuous factor map of $X_{\tilde{\eta }}$ has two irregular fibres modulo ${\mathbb {Z}}$, namely 0 and $\cdots 1,1,1 = -1/2$, but any subshift that is conjugate to one arising from a bijective substitution can only have one irregular fibre modulo ${\mathbb {Z}}$.

Theorem 17 tells us that to decide whether $\tilde{\theta }$ is almost bijective, we need to compute the minimal left ideals of $({\tilde{\theta }}^n)^{(+k)}$ with $1\le n \le 3$ and $0\le k \le 27$. Via automated computation, we have verified that $\tilde{\theta }$ has no bijective substitution shift factors.

## Figures and Tables

**Fig. 3:**
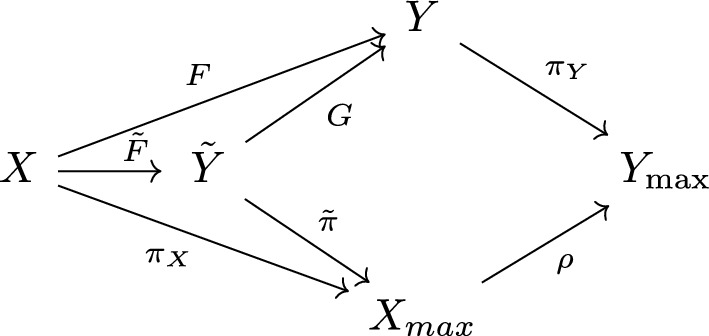
The commuting diagram for Theorem 20

## Appendix R

We first prove Theorem 4. The following lemma tells us that the assumption in Theorem 4, that $S_\theta \subset S_{\theta ^2}$, is not a deal breaker.

### Lemma 20

Let $S_\theta$ be a sub-semigroup of ${{\mathcal {F}}}({{\mathcal {A}}})$ which is generated by a family of maps $\theta \subset {{\mathcal {F}}}({{\mathcal {A}}})$. If ${{\mathcal {A}}}$ is finite then there exists $n\in {\mathbb {N}}$ such that $S_{\theta ^n} = S_{\theta ^{2n}}$ where $\theta ^n$ is the set of maps which are *n*-fold products of elements of $\theta$.

### Proof

Clearly, $S_{\theta ^2}\subset S_\theta$, and if $S_\theta = S_{\theta ^{2}}$ then also $S_{\theta ^{2}}=S_{\theta ^{4}}$. Hence, the infinite chain

$$\begin{aligned} S_\theta \supset S_{\theta ^2}\supset S_{\theta ^4}\supset \cdots \end{aligned}$$

must stabilise, as $S_\theta$ is finite. $\square$

We use the following results.

### Theorem 18

([16, Theorem 5]) Let $\theta$ be a primitive aperiodic length-$\ell$ substitution and $\pi _\theta :X_\theta \rightarrow {\mathbb {Z}}_\ell$ the equicontinuous factor map which maps all fixed points (or $\theta$-periodic points) to 0. Let $\tau :{{\mathcal {A}}}_\theta \rightarrow {{\mathcal {B}}}$ be a code and $Y = F_\tau (X_\theta )$. Then there is a factor map $\pi _Y\rightarrow {\mathbb {Z}}_\ell$ such that $\pi _\theta = \pi _Y\circ F_\tau$.

The following proposition follows from the recognizability of constant length substitutions, which was essentially shown by Dekking in [5].

### Proposition 19

Let $\theta$ be a primitive aperiodic length-$\ell$ substitution and $\pi _\theta :X_\theta \rightarrow {\mathbb {Z}}_\ell$ the equicontinuous factor map which maps all fixed points (or $\theta$-periodic points) to 0. If $x,x'\in X_\theta$ satisfy $\pi _\theta (x)=\pi _\theta (x')$ then, for all $N\in {\mathbb {N}}$ there exists $n\in {\mathbb {Z}}$ such that $\sigma ^n(x)$ and $\sigma ^n(x')$ belong to $\theta ^N(X_\theta )$. Here $\theta$ is viewed as a map on $X_\theta$ where it is injective, but not surjective.

Recall that $S_\theta$ is the sub-semigroup of ${{\mathcal {F}}}({{\mathcal {A}}})$ generated by the column maps of the substitution. Recall that a partition ${{\mathcal {P}}}$ of ${{\mathcal {A}}}$ defines a code $\tau :{{\mathcal {A}}}\rightarrow {{\mathcal {B}}}={{\mathcal {P}}}$, $\tau (a)$ is the member $A\in {{\mathcal {P}}}$ to which *a* belongs. If for each $f\in S_\theta$ and for each $A\in {{\mathcal {P}}}$ there is $A'\in {{\mathcal {P}}}$ with $f(A) \subset A'$, then the code defines an inner encoded substitution $(\eta ,\tau )$. If not, we can define a finer partition ${\tilde{{{\mathcal {P}}}}}$ which has this property, notably through the equivalence relation $a\sim b$ if for each $f\in S_\theta$, $\tau (f(a))=\tau (f(b))$. We denote the associated inner encoded system by $(\eta ,\beta )$. The alphabet ${{\mathcal {A}}}_\eta =\beta ({{\mathcal {A}}})$ of $\eta$ sits in between ${{\mathcal {A}}}$ and ${{\mathcal {B}}}$, because $a\sim b$ means that in particular $\tau (a) = \tau (b)$. Hence $\tau$ factorises $\tau = \tau '\circ \beta$ where $\tau ':\beta ({{\mathcal {A}}})\rightarrow {{\mathcal {B}}}$ is a code. In what follows we use the fact that $|{{\mathcal {A}}}|(|{{\mathcal {A}}}|-1)$ is the number of unequal ordered pairs in ${{\mathcal {A}}}$.

### Lemma 21

Let $\theta$ be a primitive aperiodic length-$\ell$ substitution over ${{\mathcal {A}}}$. Let $N=|{{\mathcal {A}}}|(|{{\mathcal {A}}}|-1)$. Suppose that $S_\theta \subset S_{\theta ^2}$. Let $a,b\in {{\mathcal {A}}}$. If $\tau (\theta ^N(a))=\tau (\theta ^N(b))$ then $\forall f\in S_\theta$ we have $\tau (f(a))=\tau (f(b))$.

### Proof

Any $f\in S_\theta$ has the form $\theta _{i_n}\cdots \theta _{i_1}$ for some *n*. Let, for $j\le n$, $f^{(j)} = \theta _{i_j}\cdots \theta _{i_1}$ and $f^{(0)}={{\textbf{1}}}$. If $f(a)\ne f(b)$ then $f^{(j)}(a)\ne f^{(j)}(b)$ for all $j\le n$ and so, if $n\ge N$ then the sequence of unequal pairs $(f^{(j)}(a),f^{(j)}(b))_{0\le j \le n}\subset {{\mathcal {A}}}^2$ hits at least one point twice, say $(f^{(j_1)}(a),f^{(j_1)}(b))=(f^{(j_2)}(a),f^{(j_2)}(b))$. Let

$$\begin{aligned} {{\tilde{f}}} = \theta _{i_n}\cdots \theta _{j_2}\theta _{j_1-1}\cdots \theta _{i_1} \end{aligned}$$

that is, leave out $\theta _i$ for $i=j_1,\cdots j_2-1$ in the product for *f*. Iterating if necessary ${{\tilde{f}}}\in S_\theta$ has at most *N* factors $\theta _i$. Furthemore, ${{\tilde{f}}}(a) = f(a)$ and ${{\tilde{f}}}(b)=f(b)$. Now $S_\theta \subset S_{\theta ^2}$ implies that we can write ${{\tilde{f}}}$ as a product of *N* factors. Therefore there is *m* such that ${{\tilde{f}}} = {\theta ^N}_m$. It follows that $\tau (\theta ^N(a))=\tau (\theta ^N(b))$ implies $\tau ({{\tilde{f}}}(a))=\tau ({{\tilde{f}}}(b))$ implies $\tau (f(a))=\tau (f(b))$. $\square$

### Proof of Theorem 4

Given the code $\tau :{{\mathcal {A}}}\rightarrow {{\mathcal {B}}}$ we have seen above that we can construct an inner encoding $(\eta ,\beta )$ of $\theta$ and $\tau = \tau '\circ \beta$. It remains to show that $F_{\tau '}:X_\eta \rightarrow Y\subset {{\mathcal {B}}}^{\mathbb {Z}}$ is injective. Clearly $X_\eta = F_{\beta }(X_\theta )$. Let ${{\tilde{x}}},{{\tilde{y}}}\in X_\eta$, so ${{\tilde{x}}}=F_{\beta }(x)$ and ${{\tilde{y}}} = F_{\beta }(y)$ for some $x,y\in X_\theta$, and suppose that $F_{\tau '}({{\tilde{x}}}) = F_{\tau '}({{\tilde{y}}})$. Hence $F_\tau (x) = F_\tau (y)$. By Theorem 18, $\pi _\theta (x)=\pi _Y(F_\tau (x)) =\pi _Y( F_\tau (y))=\pi _\theta (y)$. Lemma 19 implies that there is *n* such that $\sigma ^n(x),\sigma ^n(y) \in \theta ^N(X_\theta )$. Let $x',y'$ be pre-images, $\theta ^N(x') = \sigma ^n(x)$ and $\theta ^N(y') = \sigma ^n(y)$. We thus have $F_\tau (\theta ^N(x'))=F_\tau (\theta ^N(y'))$ which means that, $\tau (\theta ^N(x'_k))=\tau (\theta ^N(y'_k))$ for all *k*. Lemma 21 implies that $\tau (f(x'_k))=\tau (f(y'_k))$, for all *k*. Hence $\beta (x'_k)=\beta (y'_k)$, for all *k*. As $\theta$ maps members of the partition defined by $\beta$ into members of that partition we have $\beta (\theta (x'_k))=\beta (\theta (y'_k))$ hence, by iteration, $\beta (x_k)=\beta (y_k)$ for all *k*. Hence ${{\tilde{x}}}={{\tilde{y}}}$. $\square$

Next, we give a proof of Proposition 5. It is based on the proof of [4, Proposition 3.19], where there is a similar result bounding the radius of an automorphism of a constant length substitution shift. Here, we bound the radius of a factor map of a constant length substitution shift.

### Proof of Proposition 5

Let *l*, *r* be the left, right radius of *F* respectively, and let $n=(-l,r)$. Then there is a code $\tau :{{\mathcal {A}}}_{\theta ^{(n)}}\rightarrow {{\mathcal {B}}}$ such that $F_\tau :X_{\theta ^{(n)}}\rightarrow Y$ is a factor map and such that $F = F_\tau \circ F_{\imath }^{-1}$. Note that by Lemmas 1 and 2, $F_{\imath }^{-1}$ maps fixed points to fixed points.

To prove the second statement, suppose that $Y=X_\eta$ for some length-$\ell$ substitution $\eta$ and that *F* preserves the fibre of fixed points. Let $R = \max \{ l, r \}$. We know that $(F (x))_i$ is determined by $x_{[i - R, i + R]}$, and thus $(F (x))_{[0, \ell ^n)}$ is determined by $x_{[- R, \ell ^n + R)}$. Thus, if we choose $n \geqslant \lceil \log _{\ell } R \rceil$, this ensures $R \leqslant \ell ^n$, hence $(F (x))_{[0, \ell ^n)}$ is entirely determined by $x_{[- \ell ^n, 2 \ell ^n)}$.

Fix $n \geqslant \lceil \log _{\ell } R \rceil$. Since *F* sends fixed points to fixed points, then $F (\theta ^n (X_{\theta })) \subseteq \eta ^n (X_{\eta })$. Define $G :X_{\theta } \rightarrow X_{\eta }$ as $G:= \eta ^{- n} \circ F \circ \theta ^n$. Note that $F \circ \theta ^n$ maps $X_{\theta }$ to $\eta ^n (X_{\eta })$, and the map $\eta ^{- n} :\eta ^n (X_{\eta }) \rightarrow X_{\eta }$ is well-defined by recognisability of $\eta$, so *G* is also well-defined. It is not hard to check that *G* is continuous and $G \circ \sigma = \sigma \circ G$, so *G* is also a factor map.

Given knowledge of $x_{[- 1, 1]}$, we know $(\theta ^n (x))_{[- \ell ^n, 2 \ell ^n)}$ and hence $(F \circ \theta ^n (x))_{[0, \ell ^n)}$ is also determined, as discussed above.

Recall that a substitution $\eta$ is *injective* if the map $\eta :{{\mathcal {A}}}\rightarrow {{\mathcal {A}}}^\ell$ is injective. If $\eta$ is injective, then $(F \circ \theta ^n (x))_{[0, \ell ^n)}$ determines $(\eta ^{- n} \circ F \circ \theta ^n (x))_0$, and thus *G* has left and right radius at most 1. Also, *G* must send fixed points to fixed points. If $\eta$ is non-injective on letters, we can replace it with an injectivisation $\tilde{\eta }$ of $\eta$, which is the standard example of an inner encoding of $\eta$, with two letters identified if and only if their images under $\eta$ are equal. As the associated conjugacy $X_{\eta } \rightarrow X_{\tilde{\eta }}$ has radius 0, this will not change the desired result, that *G* has left and right radius at most 1.

It remains to show that the radius restriction for *G* implies the same restriction for *F*. Minimality implies that any factor map $$X_{\theta } \rightarrow X_{\eta }$$ is entirely determined by the image of a single point. As the fixed point fibres of substitutions are finite, there can only be finitely many factor maps $$F_i:X_\theta \rightarrow X_\eta $$ which preserve the fixed point fibre. Let $${{\mathcal {F}}}:=\{ F_1, \ldots , F_{k} \}$$ be the collection of those. Let $$l_i, r_i$$ be the left and right radius of each $$F_i$$. If we define $$R = \max \{ l_1, r_1, \ldots , l_{k}, r_{k} \}$$ and take $$n \geqslant \lceil \log _{\ell } R \rceil $$, then the above argument shows that $$ \eta ^{- n} \circ F_i \circ \theta ^n$$ preserves the fixed point fibre of $$\theta $$ and hence the map $${{\mathcal {F}}}\rightarrow {{\mathcal {F}}}$$ given by $$F_i \mapsto \eta ^{- n} \circ F_i \circ \theta ^n$$ is well-defined. This map is a bijection. For, if $$ \eta ^{- n} \circ F_i \circ \theta ^n = \eta ^{- n} \circ F_j \circ \theta ^n $$, then $$F_i \circ \theta ^n (u) = F_j \circ \theta ^n (u) $$ for any point $$u \in X_{\theta }$$, and in particular for any fixed point *u* of $$\theta $$, so that $$F_i (u) = F_j (u) $$ for *u* a fixed point. Now minimality implies that the map is injective and since $${{\mathcal {F}}}$$ is finite it is also surjective.

To conclude, we have seen above that every factor map of the form $\eta ^{- n} \circ F_i \circ \theta ^n$ has left and right radius at most 1 and so the same must hold for all $F_i$. $\square$

## Appendix M

We formulate a theorem of Martin in a slightly more general way and provide the proof for the convenience of the reader [15, Thm.8.11].

### Theorem 20

Let (*Y*, *S*) be an almost automorphic factor of (*X*, *T*). Then there exists a factor $({{\tilde{Y}}},{{\tilde{S}}})$ of (*X*, *T*) which is both an extension of (*Y*, *S*) and an almost one-to-one extension of the maximal equicontinuous factor of (*X*, *T*). If (*Y*, *S*) is a proper almost automorphic factor of (*X*, *T*) then $({{\tilde{Y}}},{{\tilde{S}}})$ is also a proper almost automorphic factor of (*X*, *T*). If the factor map $F:X\rightarrow Y$ is isometric then the factor map ${{\tilde{F}}}:X\rightarrow {{\tilde{Y}}}$ is also isometric.

### Proof

See Fig. 3. Let $\pi _X:X\rightarrow X_{max}$ be the maximal equicontinuous factor map. Define the equivalence relation on *X* by $x_1\sim x_2$ if $F(x_1)=F(x_2)$ and $\pi _X(x_1)=\pi _X(x_2)$. Let ${{\tilde{Y}}} = X/\sim$ and let ${{\tilde{F}}}:X\rightarrow {{\tilde{Y}}}$ denote the canonical surjection. Clearly $\pi _X$ factors $\pi _X={\tilde{\pi }} \circ {{\tilde{F}}}$, and $F:X\rightarrow Y$ factors $F=G\circ {{\tilde{F}}}$. In particular ${{\tilde{Y}}}$ is an extension of *Y*. Let $Y_{max}$ be the MEF of *Y* with factor map $\pi _Y$. By maximality, $X_{max}$ factors onto $Y_{max}$ and we can arrange the factor map $\rho :X_{max}\rightarrow Y_{max}$ in such a way that $\pi _Y\circ F = \rho \circ \pi _X$.

We claim that ${\tilde{\pi }}:{{\tilde{Y}}}\rightarrow X_{max}$ is almost one-to-one: Let $\eta \in Y_{max}$ such that $\pi _Y^{-1}(\eta )$ contains a single element, say *y*. Let $\xi \in \rho ^{-1}(\eta )$. Let $x\in \pi _X^{-1}(\xi )$. Then $\pi _Y\circ F(x) = \eta$, hence $F(x) = y$. Hence any two elements $x_1,x_2\in \pi _X^{-1}(\xi )$ satisfy $F(x_1)=F(x_2)$ and $\pi _X(x_1)=\pi _X(x_2)$ so that ${{\tilde{F}}}(x_1)={{\tilde{F}}}(x_2)$. It follows that ${\tilde{\pi }}^{-1}(\xi )$ contains only one element.

We claim that $({{\tilde{Y}}},{{\tilde{S}}})$ is not equicontinuous. Indeed, if it were equicontinuous then *Y* would also be equicontinuous, as it is a factor of ${{\tilde{Y}}}$.

Finally, if $F=G\circ {{\tilde{F}}}$ is isometric then ${{\tilde{F}}}$ must also be isometric. $\square$

## Appendix J

We recall some background material on subsemigroups of the semigroup ${{\mathcal {F}}}(X)$ of maps from *X* to itself; see also [3, 9, 17]. Here *X* is just a set and the semigroup product is composition of functions. For our purposes *X* will be a finite set. We denote by ${{\mathcal {P}}}(X)$ the set of subsets of *X*.

Let $f:X\rightarrow Y$. We denote by $f^{-1}:{{\mathcal {P}}}(Y)\rightarrow {{\mathcal {P}}}(X)$ the pre-image map but simply write $f^{-1}(y)$ for $f^{-1}(\{y\})$. The map *f* defines an equivalence relation as $x\sim x'$ if $f(x)=f(x')$. We denote the associated partition by ${{\mathcal {P}}}_f$, that is,

$$\begin{aligned} {{\mathcal {P}}}_f =\{f^{-1}(y): y\in Y\}. \end{aligned}$$

The cardinality of ${{\mathcal {P}}}_f$ equals the rank of *f*, that is the cardinality of its image $\textrm{im}f$.

Recall two of Green’s equivalence relations ${{\mathcal {L}}}$, ${{\mathcal {R}}}$. They give a first way to approach and organise a semigroup *S*. We say that $a,b\in S$ are ${{\mathcal {L}}}$-related, or ${{\mathcal {R}}}$-related, if they generate the same left, or right ideal, respectively. If *S* is a group, then these relations coincide with the full relation. Each of the above relations partition the semigroup. ${{\mathcal {R}}}$ is a left congruence and therefore $S/{{\mathcal {R}}}$ a left-*S*-module. ${{\mathcal {L}}}$ is a right congruence and therefore $S/{{\mathcal {L}}}$ a right-*S*-module.

### Lemma 22

Let *S* be a semigroup of ${{\mathcal {F}}}(X)$. If $f,g\in S$ are ${{\mathcal {R}}}$-related then $\textrm{im}f = \textrm{im}g$. If $f,g\in S$ are ${{\mathcal {L}}}$-related then ${{\mathcal {P}}}_f = {{\mathcal {P}}}_g$.

### Proof

Let *f* and *g* be ${{\mathcal {R}}}$-related, that is, $f=g$ or there is $f',g'$ such that $f = gg'$, $g=ff'$. Then clearly $\textrm{im}f\subset \textrm{im}g$ and $\textrm{im}g\subset \textrm{im}f$.

Let *f* and *g* be ${{\mathcal {L}}}$-related, that is, $f=g$ or there is $f',g'$ such that $f = g'g$, $g=f'f$. Then $f^{-1}(x) = g^{-1}({g'}^{-1}(x))$ showing that the members of the partition ${{\mathcal {P}}}_f$ are unions of members of the partition of ${{\mathcal {P}}}_g$, i.e. the partition ${{\mathcal {P}}}_g$ is finer than ${{\mathcal {P}}}_f$. A symmetric argument shows that ${{\mathcal {P}}}_f={{\mathcal {P}}}_g$. $\square$

An element $f\in S\subset {{\mathcal {F}}}(X)$ is *completely regular* if there exists $g\in S$ such that $fgf=f$ and $fg=gf$. This implies that *fg* is an idempotent. As normal inverses are unique we call *fg* the idempotent associated to *f* and denote it $f^0$.

A *completely simple* semigroup is a semigroup which has no proper bilateral ideals and which contains an idempotent. The *kernel* of a semigroup, if it exists, is its smallest bilateral ideal. If *X* is finite then any sub-semigroup of ${{\mathcal {F}}}(X)$ admits a kernel. We denote the kernel of *S* by $\ker S$; if it contains an idempotent it is completely simple, and any element is completely regular.

### Lemma 23

Let *X* be a finite set and $S\subset {{\mathcal {F}}}(X)$. Then *S* is completely simple if and only if all its functions have the same rank. Moreover, if $f,g\in S$ belong to the same right ideal, then $g^0 f = f$, while if *f*, *g* belong to the same left ideal, then $f = f g^0$.

### Proof

Given $n\in {\mathbb {N}}$, the subset of functions of rank $\le n$ form a bilateral ideal in *S*. Hence the condition that all functions have the same rank is necessary for simplicity.

Now suppose that all functions of *S* have the same rank. Let $f\in S$. Then $\textrm{im}f = \textrm{im}f^k$ for all $k\ge 1$. As *X* is finite there must by a $k>0$ such that $f = f^{k+1}$. Hence $f^{k}=f^0\in S$. The restriction of $f^0$ to $\textrm{im}f$ is the identity. Let also $g\in S$. As $fg\in S$ the argument above shows that there is $k>0$ such that the restriction of $(gf)^k$ to $\textrm{im}gf = \textrm{im}g$ is the identity. It follows that $(gf)^k g = g$. Hence *g* belongs to the bilateral ideal generated by *f*. Choosing $f\in \ker S$ we see that $S=\ker S$ and so is simple.

Suppose that $f,g\in S$ belong to the same right ideal. Then $f = g g'$ for some $g'\in S$. Hence $g^0f = g^0 g g' = g g' = f$. Similarily, if $f,g\in S$ belong to the same left ideal. Then $f = g' g$ for some $g'\in S$. Hence $f g^0 = g' g g^0 = f$. $\square$

### Corollary 8

Let *X* be a finite set and $S\subset {{\mathcal {F}}}(X)$. The kernel of *S* is given by its functions of minimal rank. It is completely simple.

The Rees structure theorem ( [3, Theorem 3.5] or [12, Theorem 2.1]) tells us that a completely simple semigroup (without zero) is isomorphic to a matrix semigroup $S \cong I\times G\times \Lambda$ where *I* indexes the right ideals of *S*, $\Lambda$ indexes the left ideals of *S*, *G* is a group and there is an $I\times \Lambda$ matrix *M* such that multiplication in $I\times G\times \Lambda$ is defined as

$$\begin{aligned}(i,g,\lambda )(i',g',\lambda ')= (i,gM_{\lambda , i'}g', \lambda ') \end{aligned}$$

The right ideals are given by $\{i\}\times G\times \Lambda$, $i\in I$, and the left ideals by $I\times G\times \{\lambda \}$, $\lambda \in \Lambda$. In particular one sees that any right ideal intersects any left ideal non-trivally.

### Proposition 21

Let $S\subset {{\mathcal {F}}}(X)$ be a completely simple semigroup and $f,g,\in S$. The following are equivalent.

1. *f* and *g* are ${{\mathcal {R}}}$-related.
2. *f* and *g* belong to the same right ideal.
3. *f* and *g* have the same image.

Moreover, the following are equivalent.

1. *f* and *g* are ${{\mathcal {L}}}$-related.
2. *f* and *g* belong to the same left ideal.
3. *f* and *g* define the same partition.

### Proof

A completely simple semigroup (without zero) is the disjoint union of its right ideals and all the right ideals are simple. As the ${{\mathcal {R}}}$-class of any element of a simple right ideal is that right ideal, we have equivalence between R1 and R2. It remains to show that R3 implies R2: Suppose that *f* and *g* belong to the same left ideal. Then $f g^0 = f$. If also $\textrm{im}f = \textrm{im}g$ then $g^0 f = f$, hence *f* and *g* belong to the same right ideal. Now suppose that *f* and *g* are arbitrary elements satisfying $\textrm{im}f=\textrm{im}g$. Then there is $h\in S$ which is in the same right ideal as *g* and the same left ideal as *f*. The first property implies $\textrm{im}h=\textrm{im}g$ and, by the above, *h* is also in the same right ideal as *f*. Hence *f* and *g* belong to the same right ideal.

The equivalence between L1 and L2 is shown as for right ideals. We show that L3 implies L2: Suppose that *f* and *g* belong to the same right ideal. Then $g^0 f = f$. If also ${{\mathcal {P}}}_f = {{\mathcal {P}}}_g$ then $f g^0 = f$, hence *f* and *g* belong to the same left ideal. The rest of the argument is as for right ideals. $\square$

### Lemma 24

Let *S* be a semigroup which admits a kernel. Let $\Phi :S\rightarrow T$ be an epimorphism onto another semigroup *T*. The restriction of $\Phi$ to the kernel of *S* is an epimorphism onto the kernel of *T*. Morever, if *S* has a unique minimal left ideal then also *T* has a unique minimal left ideal.

### Proof

Any epimorphism preserves ideals: We show this for left ideals. Let *I* be a left ideal of *S* and $t\in T$; we ought to show that $t\Phi (I)\subset \Phi (I)$. Since $\Phi$ is onto there is $s\in S$ such that $\Phi (s)=t$. Then $t\Phi (I)=\Phi (sI) \subset \Phi (I)$.

Furthermore, the preimage of an ideal is an ideal: We show this for left ideals. Let *I* be a left ideal of *T* and $s\in S$; we ought to show that $s\Phi ^{-1}(I)\subset \Phi ^{-1}(I)$. Indeed, $\Phi (s\Phi ^{-1}(I)) = t I \subset I$.

Now the above implies that $I:=\Phi (\ker S)$ is the kernel of *T*: Indeed, by the first paragraph it is a bilateral ideal. Let $J\subset I$ be a bilateral ideal of *T*. Then $\Phi ^{-1}(J)$ is a bilateral ideal in *S* by the second paragraph. $\Phi ^{-1}(J)$ thus contains $\ker S$. Hence $\Phi (\ker S)\subset J$ showing that $J=I$.

Suppose now that $\ker S$ is left simple and that $L_1,L_2$ are left ideals of $\ker T$. Then $\Phi ^{-1}(L_1)\cap \ker S$ and $\Phi ^{-1}(L_2)\cap \ker S$ belong to the same left ideal. As epimorphisms preserve ideals their image under $\Phi$ belongs to the same left ideal. Hence $L_1$ and $L_2$ belong to the same left ideal. Hence $L_1=L_2$. $\square$

## Footnotes

1 Here we follow the terminology of de Vries [6], which sometimes diverges from that of Veech, who calls any almost one-to-one extension an almost automorphic extension.
